# The effects of sex and gender attributes on clinical outcomes: a systematic review

**DOI:** 10.1186/s13293-025-00772-x

**Published:** 2025-12-29

**Authors:** Anisa Brar, Anjali Issar, Thaisa Tylinski Sant’Ana, Tatyana Mollayeva

**Affiliations:** 1 Department of Occupational Science and Occupational Therapy, University of Toronto, Toronto, ON Canada; 2 KITE Toronto Rehabilitation Institute, University Health Network, Toronto, ON Canada

**Keywords:** Androgyny, Biological sex, Clinical outcomes, Femininity, Gender and sex assessment, Health, Masculinity, Sex hormones, Sociocultural gender

## Abstract

**Background:**

Biological sex and sociocultural gender may influence changes in health status critical to clinical decision-making, yet scientific evidence of their effects on clinically relevant outcomes remain uncertain. We aimed to systematically review research on sex and gender effects on clinical outcomes and to assess the consistency and significance of associations between sex, gender, and clinical outcomes.

**Methods:**

We searched Medline, Embase, PsycInfo, CINAHL, and Web of Science from each database’s inception to November 20, 2023, and included English language peer-reviewed research utilizing standardized measures of sex and gender attributes in adults to measure their association with clinically relevant outcomes. We performed a risk of bias assessment and certainty assessment using criteria set a priori. We created visualizations of results with links to study quality and sex and gender attributes, which facilitated certainty assessment. We reported results across sex and gender-related attributes and measures.

**Results:**

Of the 12,964 unique records identified, 19 studies with a total of 643,093 participants (54% male) were included in data synthesis. Four studies measured attributes of sex (testosterone, sex-specific polygenic score), and 15 studies measured attributes of gender (gender identity, roles, and adherence to masculine norms). We observed great heterogeneity in the direction and significance of the associations, resulting in evidence of moderate certainty only for the association between testosterone level and depression, and erectile function. We regarded all other evidence as very low in certainty.

**Conclusion:**

Research findings regarding the effects of sex and gender attributes on clinical outcomes is variable. However, results suggest that neither sex nor gender attributes should be ignored when investigating clinically relevant outcomes. To enhance certainty, future research should delve into sex and gender attributes concurrently, taking into account that clinical disorders are not evenly distributed across sexes and genders. This approach would provide needed evidence to drive precision medicine and person-centered care.

**PROSPERO:**

CRD42023456917. Funding: Global Brain Health Institute, Alzheimer’s Association, and Alzheimer’s Society UK Pilot Award for Global Brain Health Leaders (GBHI ALZ UK-23–971123); Canada Research Chairs Program for Neurological Disorders and Brain Health (CRC-2021-00074).

**Supplementary Information:**

The online version contains supplementary material available at 10.1186/s13293-025-00772-x.

## Highlights

Past reviews explored the effect of sex and gender on clinically relevant outcomes in adults; however, their focus was only on binary sex or gender.

To address the gap, we examined the effect of sex and gender attributes collected through standardized measurements in 643,093 adults on clinically relevant outcomes.

We presented a comprehensive synthesis of associations between sex and gender attributes and 34 clinically relevant outcomes across multiple domains, including cardiovascular health, mental health, endocrine and metabolic health, reproductive and hormonal health, and sexual health, among others.

We regarded evidence on the association between testosterone level and depression, and erectile function, as moderate in certainty; all other evidence was very low in certainty.

The results of our review have important implications for future research advancing precision medicine and person-centered care.

**Supplementary Information:**

The online version contains supplementary material available at 10.1186/s13293-025-00772-x.

## Introduction

Studies of the effects of biological sex and sociocultural gender in health and disease have been ongoing for decades [1, 2]. However, the meaning and value of these effects have been questioned, in part because quantifying the diverse attributes that comprise biological sex and sociocultural gender is complex, evolves over time, and remains challenging to capture despite the strong scientific demand to use numerical measurement in medicine and scientific research. As Lord Kelvin famously stated in 1883, “when you cannot express it in numbers, your knowledge is of a meagre and unsatisfactory kind” [3, 4]. It has also been argued that “the ability to measure a variable, no matter how indirectly, is dependent on one’s ability to define it. Unless we know what a term means, we can’t show that it exists” [5].

The Canadian Institutes of Health Research (CIHR) defines sex as a biological construct associated with physical and physiological characteristics, including chromosomes, hormones, and anatomical features, and gender as a sociocultural construct determined by social roles, behaviors, expressions, and identities [6]. The constructs of sex and gender are not independent; they influence and shape each other, impacting health and disease outcomes of people, families, and societies at large [7].

Several reviews have explored the effect of binary sex and gender on clinical outcomes, but have reported inconsistent results [8–15]. These discrepancies likely reflect heterogeneity in the biological sex and sociocultural characteristics of research participants within the reported binary sex and gender concepts, as well as selective samples of people with specific clinical conditions. In addition, it is not known if the meaning of gender attributes would be the same in people with differences in clinical outcomes, or those of different sexes. To our knowledge, the specific attributes of sex and gender and their associations with clinical outcomes have not been systematically reviewed. We conducted a systematic review of attributes of sex and gender in adult persons with three goals: (1) to identify and critically appraise studies that used standardized measurements to capture the effects of sex and/or gender attributes on clinically relevant outcomes; (2) to categorize sex and gender attributes, their related measures, and measures of outcomes; and (3) to examine the relevance and certainty of the associations between sex and gender attributes and clinical outcomes.

## Methods

### Protocol and registration

We followed the Preferred Reporting Items for Systematic Reviews and Meta-Analyses (PRISMA) Guideline to conduct and report our systematic review (Supplementary Material S1). We registered the protocol with the International Prospective Register of Systematic Reviews (PROSPERO, CRD42023456917) on September 2, 2023 (Supplementary Material S2).

### Search strategy

We developed a search strategy (Supplementary Material S3) in collaboration with an information specialist at a large rehabilitation research-teaching hospital (Toronto Rehabilitation Institute, University Health Network). The search strategy used a mix of keywords and subject headings (e.g. MeSH, Emtree) combined using the Boolean operators AND and OR and applied the following concepts: (A) any gender or sexuality subject headings, (B) any gender or sexuality text words in the title or author-supplied keywords, (C) any two gender or sexuality text words in the abstract (using Ovid’s frequency operator). Search terms for the concepts were sourced from a previous review [16]. We applied a search filter to each database search to exclude pediatric studies, and search filters developed by the Canadian Agency for Drugs and Technologies in Health (CADTH), to limit our searches to observational studies. We searched MEDLINE (Ovid), Embase (Ovid), PsycInfo (Ovid), Web of Science and CINAHL (EBSCOhost) from each database’s inception in 1971, 1972, 1967, and 1961, respectively, until November 20, 2023. We exported results from each database into Endnote for duplicate removal and subsequently imported results into Covidence before the screening stage. We cross-checked the references list of all included studies.

### Eligibility criteria based on PICOS framework

We defined eligibility criteria for study inclusion a priori, using the PICOS framework:

- **P** (Population): human participants older than 16 years of age, of any sex (i.e., male, female, other) and any gender (i.e., man, woman, gender-diverse).
- **I** (Intervention): this was not applicable as this was a systematic review of observational studies.
- **C** (Comparisons): standardized tools, scales, measurements, or norm-referenced values to capture attributes of sex (i.e., biological attributes in humans, including chromosomes, gene expression, hormone levels and function, and reproductive/sexual anatomy), attributes of gender (i.e., the socially constructed roles, behaviors, expressions and identities of girls, women, boys, men, and gender diverse people), or attributes of both sex and gender.
- **O** (Outcomes): any clinical outcome (i.e., outcomes related to a medical diagnosis or a sign or symptom).
- **S** (Study design): observational studies of any design (i.e., quantitative, mixed methods, cohort, cross-sectional, case control).

Human biology exists within the context of a dynamic and evolving social environment, which, in turn, is shaped by societal expectations based on one’s biological sex. In the studies included in this review, authors frequently used the terms for sex (male, female) and gender (men, women) interchangeably, without distinguishing between these constructs. In the absence of the ability to distinguish between these terms, we opted to use the terms ‘male’ and ‘female’ to maintain consistency in data synthesis and reporting. This decision was arbitrarily set, and it should not be assumed that male = man and female = woman.

### Inclusion and exclusion criteria

We included studies if they met the following criteria: (i) investigated the association between a sex and/or gender attribute of adults (i.e., mean study population age ≥ 16 years) and clinically relevant outcomes using a person- or non person reported outcome measure; (ii) the sex and/or gender measure was standardized, and used at least twice in research coming from different teams of investigators; and (iii) the research was published in English in a peer-reviewed journal. We excluded studies in which the only measure of sex/gender was the self-identification of participants’ sex/gender and studies which used a sex/gender measurement tool but did not link the scores to clinically relevant outcomes. Letters to the editor, case reports, dissertations, and studies with no primary data were excluded.

### Study selection process

At least two reviewers, including the primary authors (AB and AI), independently assessed the titles and abstracts of the identified studies against the predetermined inclusion and exclusion criteria. In the second stage, the two primary authors (AB and AI) independently assessed the full texts of potentially relevant studies to determine their compliance with the inclusion criteria. Studies that did not meet inclusion criteria were excluded (Supplementary Material S4). The senior author (TM) reviewed the quality of the first and second levels of screening. Discrepancies in inclusion/exclusion were resolved by discussion between study authors.

### Data extraction

Two reviewers (AB and AI) used a standardized data extraction sheet developed by the senior author (TM) to independently collect study characteristics and outcome data [17]. Data that were extracted included (i) study information (i.e., authors, publication year, country, location of research, objective, study design, inclusion/exclusion criteria, sample size); (ii) participant characteristics (i.e., age, sex and any other reported parameters relevant to analysis); (iii) sex and/or gender measures used, outcome measures, statistical analyses; and (iv) outcomes and key findings related to sex and gender. If the information was unclear, we planned to contact study authors to elaborate on the results and provide further details. The senior author (TM) checked the accuracy of data extraction. Inconsistencies were resolved through group discussion.

### Data synthesis

Heterogeneity across PICOS characteristics precluded numerical reporting of sex and gender effects as well as pooling of risk estimates for the sex and gender variables; as such, meta-analysis in its classic form was not appropriate. We used a best-evidence synthesis approach to organize findings by tabulation and qualitative description. We grouped studies into two main categories: (1) sex effects and (3) gender effects, and further divided by attribute and by outcome. We extracted the effect sizes of sex and/or gender associations with clinically relevant outcomes. All attributes of sex and gender, significant and non-significant, as reported by authors, were considered associations, and not causal factors.

To capture and interpret expression of sex-linked attributes and gender attributes in the results of included studies, the social variables included in the statistical analysis were monitored via PROGRESS variables; namely, place of residence, race/ethnicity/culture/language, occupation, religion, education, socioeconomic status, and social capital via family and/or other social groups. All variables studied in relation to the outcome of interest were extracted and reported in Tables 1 and 2, and Figs. 2, 3 and 4.

**Table 1:** Summary of all included studies investigating effect of sex or gender attributes on clinical outcomes

Author (year); Journal; Country; Region; City; Location of research; Study Quality (Fair, Good, Excellent)	(1) Objective (2) Design (3) Follow up/assessment times, if any (4) Inclusion criteria a. Social b. Clinical c. Behavioural d. Other (5) Exclusion criteria a. Social b. Clinical c. Behavioural d. Other	(1) Total sample size, *n* (M/F) (2) Attrition, % (if multiple assessments) (3) Age (mean ± SD) or range (4) Sex, %M (5) Other parameters reported (6) Parameters considered in analysis a. Primary predictor(s)* b. Other	(1) Measure of sex and/or gender (2) Measure of outcome(s) (3) Statistical analysis/analysis controls for	(1) Sex- and/or gender- related results (2) Other parameters related to outcome(s) (3) Researcher notes
1. Arcand M, et al. (2023) [21]; *Front Psychol*; Canada; Quebec; Montreal; Community; Fair	(1) Examine effects of sex & GR on stress, depr, anx during COVID-19 (2) Longitudinal (3) F/u at 3mos (t_1_), 6mos (t_2_), 9mos (t_3_), 12mos (t_4_) (4) a. NR b. No meds for mental illness c. NR d. NR (5) a. NR b. NR c. NR d. Incomplete gender role questionnaire	(1) 153 (50 M/103F) (2) t_1_ = 2.0%; t_2_ = 7.2%; t_3_ = 13.7%; t_4_ = 9.8% (3) M (37.65) F (32.21) (4) 33% M (5) NR (6) a. Masc/fem; binary sex b. Place of residence, occupation, education	(1) Binary sex (M, F); BSRI-SF (2) DASS-21 (3) Linear mixed effects models/binary sex, time(t_1_-t_4_); *post hoc* contrast comparisons/binary sex, time (t_1_-t_4_)	(1) No sex/gender effects on depr; F w/high fem had ↑ stress than M w/high fem at t_1_; F w/low fem had ↑ anx than M w/low fem at t_4_ (2) No effect on stress, depr, anx Sx (3) F had more Sx of stress & anx than M; no assoc b/w masc traits & stress, anx, or depr
2. Boeri L, et al. (2017) [22]; *J Sex Med;* Italy; Milan; Academic hospital; Good	(1) Examine impact of low cFT/low TT on androgen-related Sx (2) Cross-sectional (3) NA (4) a. Male, heterosexual, sexually active, European Caucasian b. ED as primary complaint c. NR d. NR (5) a. NR b. Hx of radical prostatectomy, radical cystectomy, pelvic radiation therapy, androgen deprivation, TD/T therapy c. NR d. NR	(1) 500 (500 M/0F) (2) NA (3) 18–70 (4) 100% M (5) Race (100% European Caucasian), age, BMI, waist circumference, CCI score, smoking, hypertension, MetS (6) a. TT & cFT levels b. Age, BMI, CCI score	(1) Normal, low cFT; normal, low TT (2) IIEF; BDI (3) One-way ANOVA; Pearson χ^2^ test; univariable, multivariable linear regression analyses/age, BMI, CCI score	(1) TT_norm_+cFT_low_, TT_low_+cFT_low_ had ↓ IIEF-EF, IIEF-SD, IIEF-OF, ↑ BDI (2) Age inversely assoc w/IIEF-EF, IIEF-SD, IIEF-OF (3) Low cFT values indep predicted IIEF-EF, IIEF-SD, IIEF-OF, BDI, irrespective of TT level
3. Cunningham ML, et al. (2020) [23]; *Body Image*; Australia; New South Wales; Sydney; Community; Good	(1) Examine indirect pathway linking masc discrepancy stress to MD Sx via emotion dysreg (2) Cross-sectional (3) NA (4) a. Male b. NR c. NR d. NR (5) a. NR b. NR c. NR d. NR	(1) 391 (391 M/0F) (2) NA (3) 18–50 (22.06 ± 5.34) (4) 100% M (5) Region of birth (48.3% Australia, 18.4% North America, 17.4% Asia), language (71.7% English, 13.4% Chinese), sexual orientation (6) a. Masc discrepancy stress b. NR	(1) MGRDSS (2) MDDI, DERS (3) Descriptive statistics; Pearson zero-order correlations/NR	(1) Masc discrepancy stress had sig pos assoc w/MD; Sig indirect pathway from masc discrepancy stress to MD Sx via emotion dysreg (2) NA (3) Lack of emotion reg strategies is potential pathway b/w masc discrepancy stress & MD Sx
4. Dumesic DA, et al. (2019) [24]; *J Clin Endocrinol Metab*; USA; California; Los Angeles; Academic hospital; Good	(1) Examine relationship b/w TT & adipose-IR in F w/PCOS & controls (2) Prospective cohort (3) NA (4) a. Female, non-Hispanic white b. Dx of PCOS c. NR d. NR (5) a. NR b. Congenital adrenal hyperplasia, thyroid dysfunction, hyperprolactinemia c. NR d. NR	(1) 28 (0 M/28F) (2) NA (3) 19–35 (4) 0% M (5) Race (100% non-Hispanic white) (6) a. TT & free T b. NR	(1) Continuous TT (ng/dL) & free T (pg/mL) (2) Calculated adipose-IR (3) Pearson correlation coefficient/age, obesity	(1) TT & free T levels has pos assoc w/adipose-IR in control + PCOS (2) NA (3) Pos assoc of adipose-IR w/androgen levels suggests in vivo effect of androgens on adipocyte function
5. Gibson PA, et al. (2016) [25]; *J Affect Disord*; USA; Community; Good	(1) Investigate relationship b/w sex, gender, education on depr in young adults (2) Cross-sectional (3) NA (4) a. NR b. NR c. In current heterosexual relationship d. Data from National Longitudinal Survey of Adolescent Health Wave III (5) a. NR b. NR c. NR d. NR	(1) 4302 (1742 M/2460F) (2) NA (3) 18–26 M (22.28 ± 1.68) F (21.95 ± 1.74) (4) 40% M (5) Education, race, family status in adolescence, parental education (6) a. Masc/fem; binary sex b. Education	(1) BSRI (2) CES-D (3) Negative binomial regression/Race, age, family status in adolescence, parental education, adolescent alcohol use	(1) Fem in F & M was negatively assoc w/depr; M w/high masc had ↓ depr Sx; F w/high masc showed NS assoc w/depr (2) F w/high masc, non-college educated had ↑ depr Sx; college-educated had ↓ depr Sx; M w/high fem, college-educated had ↓ depr Sx; Other assoc NS (3) In M & F, fem supported ↓ depr Sx compared to masc
6. Helgeson VS. (1991) [26]; *Psychosom Med;* USA; Colorado/New York; Denver/Long Island; Hospital; Fair	(1) Investigate relationship b/w masc & social support w/recovery from MI (2) Longitudinal (3) Follow up at 3mos, 6mos, 12mos (4) a. age ≤ 70 b. Dx of acute MI c. NR (5) a. NR b. NR c. NR d. NR	(1) 90 (70 M/20F) (2) 12mos = 3% (3) 37–70 (4) 78% M (5) Education, religion, occupation, SES (6) a. Masc b. Age, SES	(1) PAQ (2) Self-report: chest pain, health perception (3) Stepwise logistic regression analysis, stepwise multiple regression analysis/sex, Peel index, psychological distress, CHD risk factors	(1) Masc did not sig predict perceived health; masc was sig predictor of post-MI chest pain (2) No sig assoc b/w sex/age/SES & recovery (3) Spouse disclosure was most sig indep predictor of chest pain, health perception
7. Hunt K, et al. (2006) [27]; *Soc Psychiatry Psychiatr Epidemiol;* United Kingdom; Scotland; Glasgow; Community; Good	(1) Investigate relationship b/w gender & SI in three generational cohorts (1930, 1950, 1970) (2) Longitudinal (3) t_0_:1987-8, t_1_:1990-1, t_2_:1995-6, t_3_:2000-2 (4) a. NR b. NR c. NR d. Data from West of Scotland Twenty-07 Study, all cohorts (5) a. NR b. NR c. NR d. NR	(1) 2125 (960 M/1160F) (2) NR (3) Age-based cohorts at t_0_: 15, 35, 55 (4) 45% M (5) NR (6) a. Masc/fem; binary sex b. NR	(1) BSRI-SF (2) SI (3) Logistic regression models/NR	(1) In M & F, masc was assoc w/SI in 1930/1950 cohorts; fem was not assoc w/SI in any age cohorts in M or F (2) NA (3) In early, late middle age, M & F w/high masc report ↑ SI; no relationship b/w fem & SI in M or F at any age
8. Hunt K, et al. (2007) [28]; *Int J Epidemiol*; United Kingdom; Scotland; Glasgow; Community; Good	(1) Investigate relationship b/w GRO & CHD mortality (2) Prospective cohort (3) NA (4) a. NR b. NR c. NR d. Data from West of Scotland Twenty-07 Study, oldest cohort (6) a. NR b. NR c. NR d. NR	(1) 1551 (704 M/847F) (2) NA (3) 55 (4) 45% M (5) SES (6) a. Masc/fem; binary sex b. SES	(1) BSRI (2) Death from CHD (3) Univariate statistics, Cox regression models/smoking, binge drinking, BMI, systolic BP, income, psychological well-being	(1) In M w/high fem, ↓ risk of CHD mortality; in F w/high fem & in M & F w/high masc, NS relationship w/CHD mortality (2) NS change in assoc when adjusted for SES (3) Relationship b/w ↓ fem & ↑ CHD mortality in M, not F; NS assoc b/w masc & CHD mortality in M & F
9. Iwamoto D, et al. (2018) [29]; *Am J Mens Health;* USA; Maryland; College Park; Community; Good	(1) Examine relationship b/w masc norm conformity & depr Sx in young men (2) Longitudinal (3) Follow-up at 6mos (4) a. Male, 18-20yrs b. NR c. College freshman d. NR (5) a. NR b. NR c. NR d. NR	(1) 322 (322 M/0F) (2) NR (3) 18–20 (18 ± 0.38) (4) 100% M (5) Race, education (6) a. Masc b. NR	(1) CMNI-29 (2) BDI-II (3) Negative binomial regression model/NR	(1) Adherence to playboy, self-reliance, violence norms had pos assoc w/depr; adherence to winning & power over women norms had neg assoc w/depr (2) NA (3) Sig relationship b/w depr severity & distinct masc norms in young men
10. Kerr P, et al. (2021) [30]; *J Psychosom Res;* Canada; Quebec; Montreal; Community; Good	(1) Measure effect of gender roles on MH & workplace stress in psychiatric hospital workers (2) Exploratory retrospective (3) NA (4) a. NR b. NR c. Employed at psychiatric hospital d. NR (5) a. NR b. NR c. NR d. NR	(1) 192 (55 M/137F) (2) NA (3) 18–72 (40.5) (4) 29% M (5) Occupation, education, social capital (6) a. Masc/fem b. Occupation, age	(1) BSRI-SF (2) BDI-II, PTSD-CC (3) Structural equation model (path analysis)/other job strain factors	(1) Masc & fem had neg assoc w/depr; Masc had neg assoc w/trauma sx (2) Age assoc w/↓ depressive Sx & social support; pos assoc b/w occupation & psychological demands (3) Gender role endorsement assoc w/psychosocial outcomes
11. Leinonen JT, et al. (2023) [31]; *Commun med;* Finland; Community; Good	(1) Examine role of T in metabolic conditions & sex-specific Dx in both M & F (2) Cross-sectional (3) NA (4) a. NR b. NR c. NR d. Data from UK Biobank (white British subset) & FinnGen registry (5) a. NR b. NR c. NR d. NR	(1) 625,650 (2) NA (3) 24–73 (4) NR (5) NR (6) a. TT & free T, binary sex b. NR	(1) TT (nmol/L), free T (nmol/L) (2) Diseases w/links to hormones (3) PGS & MR Egger analyses/SHBG, BMI, PCOS, menopause, genetic pleiotropy	(1) In M, ↑ free T has causal relationship w/prostate cancer, ↓ osteoporosis; in F, ↑ free T has causal relationship w/hirsutism, PCOS, PMB, breast cancer, ↑ TT has causal relationship w/hirsutism, PMB, breast cancer (2) In F, T levels may have ↑ effect on disease risk, inc PCOS (3) The relationship b/w T levels & metabolic & endocrine traits is highly complex
12. Möller-Leimkühler A, et al. (2009**) **[32]; *J Affect Disorders;* Germany; Munich; School; Good	(1) To assess relationship b/w M depr in university students w/sex, GRO, personality traits (2) Cross-sectional (3) NA (4) a. University students b. NR c. NR d. Recruited from different faculties at the Ludwig-Maximilians-University of Munich (5) a. NR b. NR c. NR d. NR	(1) 1018 (518 M/500F) (2) NA (3) M (24.51) F (23.66) (4) 51% M (5) Age, education (6) a. Masc/fem; binary sex b. NR	(1) GE-PAQ (2) WHO-5, GSMD (3) Pearson Correlation analysis/NR; Multivariate ANOVA/binary sex, GRO; Principal Component Analysis/NR	(1) Risk of M depr was sig ↑ in F, not M (2) Binary sex, GRO had indep effects on risk of M depr w/o interacting effects (3) Risk of M depr ↓ in F w/↑ masc, but ↑ in F w/↓ masc
13. Nguefack H, et al. (2022) [33]; *Frontier Pain Res*; Canada; Quebec; Montreal; Community; Excellent	(1) Explore the assoc b/w GI & GR & their interactions w/participants w/CP (2) Retrospective (3) NA (4) a. Aged 18-88yrs b. NR c. NR d. Participants from COPE cohort in Quebec, Canada (5) a. NR b. NR c. NR d. NR	(1) 1343 (199 M/1119F/4NB) (2) NA (3) 18–88 (50.06 ± 13.15) (4) 15% M (5) Education, employment status, country of birth (6) a. Masc/fem b. Age, education, employment status, country of birth	(1) BSRI (2) Self-reported adverse effects of pain medication (3) Multi-variate two-part regression model/pain onset & management, location, employment, disability, education, age, health, substance use	(1) Adg participants had ↑ severe adverse effects; F had sig ↑ severe adverse effects compared to M (2) NR (3) GI, GR differences were assoc w/number of severe adverse effects
14. Po Yee Lo I, et al. (2019) [34]; *Arch Sex Behav;* United Kingdom; Oxford; USA; Louisiana; Texas; Arlington; Victoria; Abbotsford; Community; Excellent	(1) Examine effects of different types of GR on depr (2) Cross-sectional (3) NA (4) a. Female, aged 18–35yrs b. NR c. Speak & read Chinese, identifies as lesbian d. Citizen of Hong Kong (5) a. NR b. NR c. NR d. NR	(1) 438 (0 M/438F) (2) NA (3) 18–35 (24.67 ± 4.6) (4) 0% M (5) Occupation, education, relationship status, religion (6) a. Masc/fem b. NR	(1) BSRI, (2) HADS (3) ANOVA, structural equation model/age	(1) Strong masc & adg traits sig assoc w/↓ depr; Strong fem traits were sig assoc w/↑ depr (2) NR (3) ↑ masc & fem traits can promote psychological health
15. Short S, et al. (2023) [35]; *Midwifery;* United Kingdom; Community; Good	(1) Explore link b/w masc & depr in fathers in PP period (2) Cross-sectional (3) NA (4) a. 1^st-^ or 2^nd-^time father in UK b. NR c. NR d. Youngest child < 1 yr (5) a. NR b. NR c. NR d. NR	(1) 118 (118 M/0F) (2) NA (3) 1 st -time fathers (33.71 ± 6.01) 2nd -time fathers (35.56 ± 4.64) (4) 100% M (5) Sexual orientation, race (93% Caucasian, 5% Asian & Mixed Asian, 2% Black), occupation, relationship status, residence, parental leave (6) a. Masc norms b. Age, parental leave status	(1) CMNI (2) EPDS, MSPSS (3) Partial correlations/age	(1) Post-natal depr had pos correlation w/Self-Reliance & Primacy of Work (2) Parental leave status had NS effect on depr & perceived social support (3) Conformity to masc norms (Self-Reliance, Primacy of Work) was related to PP depr
16. Snyder PJ, et al. (2016) [36]; *New Engl J Med;* USA; Pennsylvania; Philadelphia; Hospital; Good	(1) Determine the impact of T tx in men w/low T levels (2) Double-blind placebo controlled (3) Assessment at 3mos, 6mos, 9mos, 12mos (4) a. Age ≥ 65yrs b. Serum T < 275ng/dL c. NR d. ↓ libido, difficulty w/walking or stairs, ↓ VT (5) a. NR b. ↑ risk of prostate cancer, CVD; Hx of prostate cancer, hypogonadism, depr; meds that alter T levels c. NR d. Non-ambulatory or physically disabled	(1) 790 (790 M/0F) (2) 12mos = 10.8% (3) ≥ 65yrs (4) 100% M (5) Race (6) a. TT & free T b. Race, age	(1) TT (ng/dL), free T (ng/dL) (2) PDQ, FACIT-Fatigue (3) Random effects models/baseline TT, age, location, med Rx	(1) Tx group had ↑ PDQ scores & ↓ in depr sx compared to placebo (2) No sig interactions of T tx w/age or race (3) ↑ of T levels in men ≥ 65yrs to the low-normal range for men 19-40yrs had sig effects on sexual function, mood
17. Vafaei A, et al. (2016) [37]; *PLOS One;* Canada; Ontario, Kingston; Quebec, Montreal Brazil; Rio de Grande Norto; Community; Excellent	(1) Assess assoc between GR & depr in older adults (2) Cross-sectional (3) NA (4) a. Aged 65–74yrs b. NR c. NR d. Patients of family medicine teams in Kingston & Saint-Hyacinthe (Canada), Tirana (Albania), Manizales (Colombia) & Natal (Brazil), (5) a. NR b. NR c. NR d. NR	(1) 1967 (942 M/1025F) (2) NA (3) 65–74 M (69.1.1 ± 2.9) F (69.1 ± 2.8) (4) 48% M (5) Education, marital Status, income (6) a. Masc/fem b. Binary sex, education, marital status, income, SRH	(1) BSRI (2) CES-D (3) Bivariate analyses/binary sex; Multi-linear analyses/binary sex, education, marital status, income, SRH, chronic conditions	(1) M & F w/masc traits had ↑ rates of depr; M & F w/adg traits had ↓ rates of depr (2) Marital status & income had indep effects on depr (3) Adg older adults reported the fewest depr Sx
18. Yang X, et al. (2018) [38]; *J Affective Disorders;* China; Hong Kong; Shenzhen; Community; Good	(1) Examine link b/w masc role discrepancy & MH (2) Cross-sectional (3) NA (4) a. Male, aged 18-60yrs b. NR c. Chinese M, Chinese speaking, reside in Hong Kong d. NR (5) a. Aged ≥ 60yrs b. NR c. NR d. NR	(1) 2000 (2000 M/0F) (2) NA (3) 18–60 (4) 100% M (5) Education, marital status (6) a. Masc b. Age, education, marital status	(1) MGRDSS (2) CESD-10, SCS (3) Pearson correlation/Age, relationship status, education	(1) MRD had pos assoc w/depr & social anx (2) Age had sig assoc w/stress & social anx (3) MRD had sig impact on depr
19. Zeldow PB, et al. (1987) [39]; *J Pers Assess;* USA; Illinois; Chicago; Community; Fair	(1) Examine relationship b/w masc & fem with adjustment, interpersonal functioning in medical school (2) Longitudinal (3) Follow up at 21mos (4) a. NR b. NR c. NR d. 1 st year medical students (5) a. NR b. NR c. NR d. NR	(1) 115 (67 M/32F) (2) 21mos = 18% (3) 25.4 (4) 58% M (5) NR (6) a. Masc/fem b. NR	(1) PAQ (2) BDI (3) Correlation test/NR	(1) Masc and fem had NS assoc w/depr (2) NR (3) Adg had ↑ risk of depr sx

In this table we have used the terms ‘sex’, ‘male’ & ‘female’ when researchers reported results based on biological attributes of their participants, regardless of the term used in the original text. The use of the term ‘primary predictor’ does not imply causality but identifies the predictor as a main factor possibly associated with an outcome

Adg, androgynous; ANOVA, analysis of variance; Anx, anxiety; BDI, Beck Depression Inventory; BMI, body mass index; BP, blood pressure; BSRI-SF, Bem Sex Role Inventory-Short Form; BSRI, Bem Sex Role Inventory; CCI, Charlson Comorbidity Index; CES-D, Center for Epidemiological Studies-Depression Scale; CESD-10, Center for Epidemiological Studies-Depression Scale, Short Form; cFT, calculated free testosterone; CHD, coronary heart disease; CI, confidence interval; CMNI, Conformity to Masculine Norms Inventory; CP, chronic pain; CVD, cardiovascular disease; DASS-21, Depression, Anxiety, and Stress Scale-21; Depr, depression; DERS-SF, Difficulties in Emotion Regulation Scale-Short Form; Dx, diagnosis; Dysreg, dysregulation; ED, erectile dysfunction; EPDS, Edinburgh Postnatal Depression Scale; F, females; FACIT-Fatigue, Functional Assessment of Chronic Illness Therapy-Fatigue Scale; Fem, femininity; GE-PAQ, German Extended Personal Attributes Questionnaire; GI, gender identity; GR, gender role; GRO, gender role orientation; GSMD, Gotland Scale for Male Depression; HADS, Hospital Anxiety and Depression Scale; HR, hazard ratio; Hx, history; IIEF-EF, International Index of Erectile Function-Erectile Function; IIEF-OF, International Index of Erectile Function-Orgasmic Function; IIEF-SD, International Index of Erectile Function-Sexual Desire; IIEF, International Index of Erectile Function; Inc, including; Indep, independent; IR, insulin resistance; M, males; Masc, masculinity; MBI, Maslach Burnout Inventory; MD, muscle dysmorphia; MDDI, Muscle Dysmorphic Disorder Inventory; MetS, Metabolic Syndrome; MGRDSS, Masculine Gender Role Discrepancy Stress Scale; MH, mental health; MI, myocardial infarction; Mod, moderate; Mos, months; MR, Mendelian randomization; MRD, masculine role discrepancy; MSPSS, Multidimensional Scale of Perceived Social Support; NA, not applicable; NB, non-binary; Neg, Negative; NR, not reported; NS, non-significant; PAQ, Personal Attributes Questionnaire; PCOS, polycystic ovary syndrome; PDQ, Psychosexual Daily Questionnaire; PGS, polygenic score; Pos, Positive; PP, post-partum; PTSD-CC, Post-Traumatic Stress Disorder-Civilian Checklist; Ref, reference; Reg, regulation; SCS, Self-Consciousness Scale; SES, socioeconomic status; SHBG, sex hormone-binding globulin; SI, suicidal ideation; Sig, significant; SRH, self-reported health; Sx, symptoms; T, testosterone; TD, testosterone deficiency; TT, total testosterone; UD, undifferentiated; VT, vitality; W/o, without; WHO-5, World Health Organization-5 Well-Being Index; Yrs, years; ↑, increased; ↓, decreased

**Table 2:** Overview of clinically relevant outcomes and associated sex and gender attributes presented in this review

Author (year) | study sample sex composition (M, F, NB)	Studied associations and results			
	Sex or gender attribute | measure	Outcome | measure | outcome category	Variables controlled for in reported associations	Sex specific associations: positive (+), negative (−), non-significant (NS)
Leinonen JT, et al. (2023) [31] | NR	Hormone level | norm-referenced free T and TT	Cardiac death | ICD-10 diagnostic codes | cardiovascular health	SHBG, BMI, PCOS, menopause, genetic pleiotropy	TT in M: NS Free T in M: NS TT in F: NS Free T in F: NS
Leinonen JT, et al. (2023) [31] | NR	Hormone level | norm-referenced free T and TT	CHD | ICD-10 diagnostic codes | cardiovascular health	SHBG, BMI, PCOS, menopause, genetic pleiotropy	TT in M: NS Free T in M: NS TT in F: NS Free T in F: NS
Hunt K, et al. (2007) [28] | 704 M, 847 F	Gender attributes | BSRI	CHD mortality | reported mortality from CHD | cardiovascular health	Smoking, binge drinking, BMI, systolic BP, SES, psychological well-being	Masculinity in M: + Undifferentiated in M: NS Femininity in M: NS Masculinity in F: NS Undifferentiated in F: NS Femininity in F: NS
Dumesic DA, et al. (2019) [24] | 0 M, 28 F	Hormone levels | norm-referenced free T and TT	Adipose insulin resistance | calculated adipose insulin resistance | endocrine and metabolic disorders	Age, obesity	cFT: + TT: +
Leinonen JT, et al. (2023) [31] | NR	Hormone level | norm-referenced free T and TT	Hypothyroidism | ICD-10 diagnostic codes | endocrine and metabolic disorders	SHBG, BMI, PCOS, menopause, genetic pleiotropy	TT in M: NS Free T in M: NS TT in F: NS Free T in F: NS
Leinonen JT, et al. (2023) [31] | NR	Hormone level | norm-referenced free T and TT	Obesity | ICD-10 diagnostic codes | endocrine and metabolic disorders	SHBG, BMI, PCOS, menopause, genetic pleiotropy	TT in M: NS Free T in M: NS TT in F: NS Free T in F: NS
Leinonen JT, et al. (2023) [31] | NR	Hormone level | norm-referenced free T and TT	Type 2 diabetes | ICD-10 diagnostic codes | endocrine and metabolic disorders	SHBG, BMI, PCOS, menopause, genetic pleiotropy	TT in M: NS Free T in M: NS TT in F: NS Free T in F: NS
Leinonen JT, et al. (2023) [31] | NR	Hormone level | norm-referenced free T and TT	Anaemia | ICD-10 diagnostic codes | hematologic disorders	SHBG, BMI, PCOS, menopause, genetic pleiotropy	TT in M: NS Free T in M: NS TT in F: NS Free T in F: NS
Nguefack H, et al. (2022) [33] | 199 M, 1119 F, 4NB	Gender attributes | BSRI	Adverse effects from pain medication | self-report | medication use	Circumstances surrounding onset of pain, pain treatment and effectiveness, pain location, employment, disability, education, age, general health score, substance use	Masculinity: NS Femininity: NS Androgyny: +
Leinonen JT, et al. (2023) [31] | NR	Hormone level | norm-referenced free T and TT	Statin use | ICD-10 diagnostic codes | medication use	SHBG, BMI, PCOS, menopause, genetic pleiotropy	TT in M: − Free T in M: NS TT in F: NS Free T in F: NS
Arcand M, et al. (2023)[21] | 50 M, 103 F	Gender attributes | BSRI-SF	Anxiety | DASS-21 | mental health conditions	Binary sex, time	Masculinity: NS Femininity: NS
Yang X, et al. (2018) [38] | 2000 M, 0 F	Masculine gender roles & norms | MGRDSS	Social anxiety | SCS | mental health conditions	Age, relationship status, education	Masculine role discrepancy stress: +
Arcand M, et al. (2023)[21] | 50 M, 103 F	Gender attributes | BSRI-SF	Depression | DASS-21 | mental health conditions	Binary sex, time	Masculinity: NS Femininity: NS
Boeri L, et al. (2017)[22] | 500 M, 0 F	Hormone level | norm-referenced cFT and TT	Depression | BDI | mental health conditions	Age, BMI, CCI	Normal TT and low cFT: + Low TT and normal cFT: NS Low TT and low cFT: +
Gibson PA, et al. (2016) [25] | 1742 M, 2460 F	Gender attributes | BSRI	Depression | CES-D | mental health conditions	Race, age, family living arrangements in adolescence, parental education, adolescent alcohol use	Masculinity in M: − Femininity in M: − Masculinity in F: NS Femininity in F: −
Iwamoto D, et al. (2018) [29] | 322 M, 0 F	Masculine gender roles & norms | CMNI-29	Depression | BDI-II | mental health conditions	NR	Playboy, self-reliance and violence: + Heterosexual presentation, risk-taking, and emotional control: NS Winning, power over women: −
Kerr P, et al. (2021) [30] | 55 M, 137 F	Gender attributes | BSRI	Depression | BDI-II | mental health conditions	NR	Masculinity: − Femininity: −
Möller-Leimkühler A, et al. (2009) | 518 M, 500 F	Gender attributes | GE-PAQ	Male-typed depression | WHO-5, GSMD | mental health conditions	NR	Positive masculinity: − Negative masculinity: + Positive femininity: − Negative femininity: +
Po Yee Lo I, et al. (2019) [34] | 0 M, 438 F	Gender attributes | BSRI	Depression | HADS | mental health conditions	Age	Masculinity: − Androgyny: − Femininity: +
Short S, et al. (2023) [35] | 118 M, 0 F	Masculine gender roles & norms | CMNI	Post-natal depression in fathers | EPDS | mental health conditions	Age, parental leave status	Self-reliance, primacy of work: + Emotional control, power over women: NS
Snyder PJ, et al. (2016) [36] | 790 M, 0 F	Hormone levels | norm-referenced free T and TT	Depression | PHQ-9 | mental health conditions	Baseline TT, age, trial site, prescribed medications	Increased serum T level: −
Vafaei A, et al. (2016) [37] | 942 M, 1025 F	Gender attributes | BSRI	Depression | CES-D mental health conditions	Binary sex, education, marital status, income, SRH, chronic conditions	Femininity: NS Androgyny: − Undifferentiated: NS
Yang X, et al. (2018) [38] | 2000 M, 0 F	Masculine gender roles & norms | MGRDSS	Depression | CESD-10 | mental health conditions	Age, relationship status, education	Masculine role discrepancy stress: +
Zeldow PB, et al. (1987) [39] | 67 M, 32 F	Gender attributes | PAQ	Depression | BDI | mental health conditions	NR	Masculinity: NS Femininity: NS
Cunningham ML, et al. (2020) [23] | 391 M, 0 F	Masculine gender roles | MGRDSS	Muscle dysmorphia | MDDI | mental health conditions	NR	Masculine role discrepancy stress: +
Arcand M, et al. (2023)[21] | 50 M, 103 F	Gender attributes | BSRI-SF	Stress | DASS-21 | mental health conditions	Binary sex, time	Masculinity: NS Femininity: NS
Kerr P, et al. (2021) [30] | 55 M, 137 F	Gender attributes | BSRI-SF	Trauma symptoms | PTSD-CC | mental health conditions	NR	Masculinity: NS Femininity: NS
Leinonen JT, et al. (2023) [31] | NR	Hormone levels | norm-referenced free T and TT	Osteoporosis | ICD-10 diagnostic codes | musculoskeletal health	SHBG, BMI, PCOS, menopause, genetic pleiotropy	TT in M: + Free T in M: NS TT in F: NS Free T in F: NS
Leinonen JT, et al. (2023) [31] | NR	Hormone levels | norm-referenced free T and TT	Stroke | ICD-10 diagnostic codes | neurological health	SHBG, BMI, PCOS, menopause, genetic pleiotropy	TT in M: NS Free T in M: NS TT in F: NS Free T in F: NS
Helgeson VS. (1991) [26] | 70 M, 20 F	Gender attributes | PAQ	Chest pain | self-reported | perceived health status	Sex, Peel index, psychological distress, CHD risk factors	Masculinity: +
Helgeson VS. (1991) [26] | 70 M, 20 F	Gender attributes | PAQ	Perceived health | self-report | perceived health status	Sex, Peel index, psychological distress, CHD risk factors	Masculinity: NS
Leinonen JT, et al. (2023) [31] | NR	Hormone levels | norm-referenced free T and TT	Benign leiomyoma | ICD-10 diagnostic codes | reproductive & hormonal health	SHBG, BMI, PCOS, menopause, genetic pleiotropy	TT in M: NS Free T in M: NS TT in F: NS Free T in F: NS
Leinonen JT, et al. (2023) [31] | NR	Hormone levels | norm-referenced free T and TT	Birth complications | ICD-10 diagnostic codes | reproductive & hormonal health	SHBG, BMI, PCOS, menopause, genetic pleiotropy	TT in M: NS Free T in M: NS TT in F: NS Free T in F: +
Leinonen JT, et al. (2023) [31] | NR	Hormone levels | norm-referenced free T and TT	Breast cancer | ICD-10 diagnostic codes | reproductive & hormonal health	SHBG, BMI, PCOS, menopause, genetic pleiotropy	TT in M: NS Free T in M: NS TT in F: + Free T in F: +
Leinonen JT, et al. (2023) [31] | NR	Hormone levels | norm-referenced free T and TT	Female infertility | ICD-10 diagnostic codes | reproductive & hormonal health	SHBG, BMI, PCOS, menopause, genetic pleiotropy	TT in M: NS Free T in M: NS TT in F: NS Free T in F: NS
Leinonen JT, et al. (2023) [31] | NR	Hormone levels | norm-referenced free T and TT	Hirsutism | ICD-10 diagnostic codes | reproductive & hormonal health	SHBG, BMI, PCOS, menopause, genetic pleiotropy	TT in M: NS Free T in M: NS TT in F: + Free T in F: +
Leinonen JT, et al. (2023) [31] | NR	Hormone levels | norm-referenced free T and TT	Irregular menstruation | ICD-10 diagnostic codes | reproductive & hormonal health	SHBG, BMI, PCOS, menopause, genetic pleiotropy	TT in M: NS Free T in M: NS TT in F: NS Free T in F: NS
Leinonen JT, et al. (2023) [31] | NR	Hormone levels | norm-referenced free T and TT	Male infertility | ICD-10 diagnostic codes | reproductive & hormonal health	SHBG, BMI, PCOS, menopause, genetic pleiotropy	TT in M: NS Free T in M: NS TT in F: NS Free T in F: NS
Leinonen JT, et al. (2023) [31] | NR	Hormone levels | norm-referenced free T and TT	Ovarian cysts | ICD-10 diagnostic codes | reproductive & hormonal health	SHBG, BMI, PCOS, menopause, genetic pleiotropy	TT in M: NS Free T in M: NS TT in F: NS Free T in F: NS
Leinonen JT, et al. (2023) [31] | NR	Hormone levels | norm-referenced free T and TT	PCOS | ICD-10 diagnostic codes | reproductive & hormonal health	SHBG, BMI, PCOS, menopause, genetic pleiotropy	TT in M: NS Free T in M: NS TT in F: + Free T in F: NS
Leinonen JT, et al. (2023) [31] | NR	Hormone levels | norm-referenced free T and TT	Post-menopausal bleeding | ICD-10 diagnostic codes | reproductive & hormonal health	SHBG, BMI, PCOS, menopause, genetic pleiotropy	TT in M: NS Free T in M: NS TT in F: + Free T in F: +
Leinonen JT, et al. (2023) [31] | NR	Hormone levels | norm-referenced free T and TT	Prostate cancer | ICD-10 diagnostic codes | reproductive & hormonal health	SHBG, BMI, PCOS, menopause, genetic pleiotropy	TT in M: NS Free T in M: + TT in F: NS Free T in F: NS
Boeri L, et al. (2017) [22] | 500 M, 0 F	Hormone level | norm-referenced cFT and TT	Erectile function | IIEF | sexual health	Age, BMI, CCI	Normal TT and low cFT: − Low TT and normal cFT: NS Low TT and low cFT: −
Snyder PJ, et al. (2016) [36] | 790 M, 0 F	Hormone level | norm-referenced free T and TT	Erectile function | IIEF | sexual health	Baseline TT, age, trial site, prescribed medications	Serum T in M: +
Boeri L, et al. (2017) [22] | 500 M, 0 F	Hormone level | norm-referenced cFT and TT	Orgasmic function | IIEF | sexual health	Age, BMI, CCI	Normal TT + low cFT: − Low TT + normal cFT: NS Low TT + low cFT: −
Snyder PJ, et al. (2016) [36] | 790 M, 0 F	Hormone level | norm-referenced free T and TT	Sexual function | PDQ | sexual health	Baseline TT, age, trial site, prescribed medications	Serum T in M: +

BDI, Beck Depression Inventory; BMI, body mass index; BP, blood pressure; BSRI-SF, Bem Sex Role Inventory-Short Form; BSRI, Bem Sex Role Inventory; CCI, Charlson Comorbidity Index; CES-D, Center for Epidemiological Studies-Depression Scale; CESD-10, Center for Epidemiological Studies-Depression Scale, Short Form; cFT, calculated free testosterone; CHD, coronary heart disease; CMNI, Conformity to Masculine Norms Inventory; DASS-21, Depression, Anxiety, and Stress Scale-21; EPDS, Edinburgh Postnatal Depression Scale; F, females; GE-PAQ, German Extended Personal Attributes Questionnaire; GSMD, Gotland Scale for Male Depression; HADS, Hospital Anxiety and Depression Scale; IIEF International Index of Erectile Function; M, males; MDDI, Muscle Dysmorphic Disorder Inventory; MGRDSS, Masculine Gender Role Discrepancy Stress Scale; NR, not reported; NS, non-significant; PAQ, Personal Attributes Questionnaire; PCOS, polycystic ovary syndrome; PDQ, Psychosexual Daily Questionnaire; PTSD-CC, Post-Traumatic Stress Disorder-Civilian Checklist; SCS, Self-Consciousness Scale; SES, socioeconomic status; SHBG, sex hormone-binding globulin; T, testosterone; TT, total testosterone; WHO-5, World Health Organization-5 Well-Being Index

### Quality and risk of bias assessment

We used previously developed standardized forms to assess study quality and risk of bias [18]. Two reviewers (AB and AI) independently assessed the quality of each study using the Quality in Prognosis Studies (QUIPS) tool [18]. The quality and risk of bias assessment comprised the following steps: (1) evaluation of six bias categories, including study design, study participation, study attrition, associated factors, outcome measures/confounding account, and analysis; (2) application of a crude score to rate whether each source of bias was “+”, “-”, or not applicable (NA); and (3) categorization of each study into the following classifications: (i) excellent (“++”) when all or most of the criteria were fulfilled (i.e., allowing at most one ‘cannot determine’ or ‘not reported’); (ii) good (“+”), when half of the criteria were fulfilled; and (iii) fair (“-”), when less than half of the criteria were fulfilled (Supplementary Material S5). We discussed disagreements regarding the risk of bias among the two reviewers to reach consensus (Supplementary Material S6). We did not exclude studies based on the quality assessment, but considered quality in the data analysis, reporting, and interpretation of studies.

### Sensitivity analysis

We conducted sensitivity analyses to examine the consistency of associations, precision, and direction of findings as recommended by the Grading of Recommendations Assessment, Development and Evaluation (GRADE) working group [19]. This approach has been used in previously published work by the senior author [20]. We visually positioned results by study outcome to evaluate the consistency of the results among the same sex and/or gender attribute, reporting significance and direction of associations (positive, negative, non-significant associations).

We conducted subgroup analyses based on risk of bias assessment, by category and type. This allowed us to evaluate the impact of study quality on the direction and consistency of the results.

### Certainty assessment

We rated the certainty of evidence based on criteria that was set a priori. We rated the certainty of the evidence as high if two or more excellent quality studies coming from different teams of investigators were concordant regarding the observed association between sex (i.e., testosterone, estrogen, etc.) or gender (i.e., femininity, masculinity, androgyny, etc.) attribute for each clinical outcome (i.e., depression, anxiety, stress, etc.) without discordant results. We assessed the certainty of evidence as moderate if two or more studies of good and/or excellent quality were concordant in their results, with a maximum of one discordant result. We assigned low certainty if at least two fair and/or good quality studies were concordant in results, with a maximum of one discordant result. In all other situations, we assessed certainty as very low.

### Missing data

In case of missing data, we followed guidelines to contact the primary author. In the case of duplicate publications and companion papers of a primary study, the protocol was to maximize the yield of information by the simultaneous evaluation of all available data. The original study took priority for inclusion.

## Results

### Search results

Our searches identified 19,538 total unique records. After the removal of duplicates, we screened 12,964 studies and, of these, 175 studies met criteria for full text review.

**Fig. 1:**
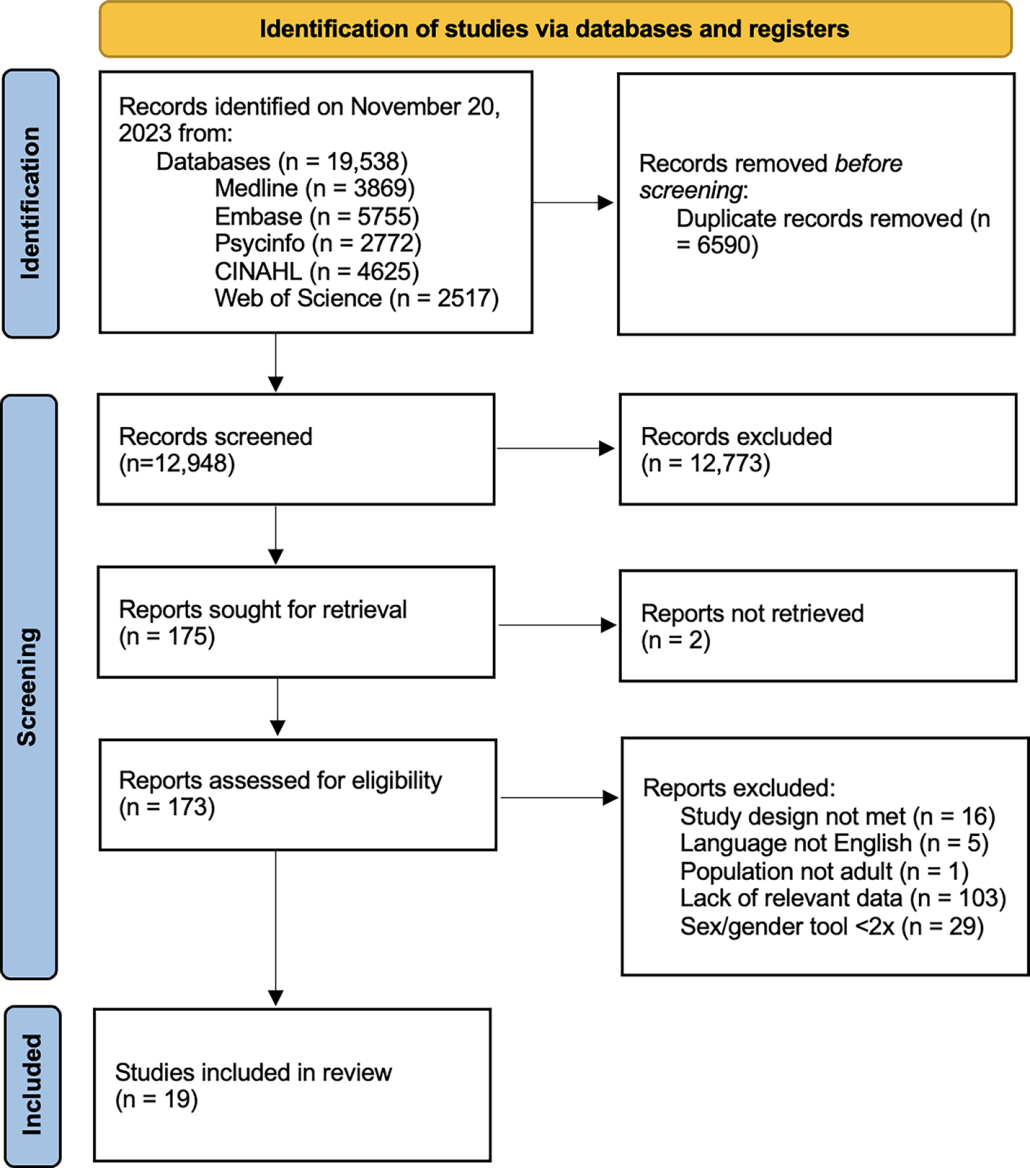
PRISMA flowchart of study selection

After full text review, we identified 19 studies which met the inclusion criteria for data collection and synthesis [21–39]. Reasons for exclusion for the remaining 156 studies were recorded (Supplementary Material S4) and are displayed in the PRISMA flow diagram (Fig. 1).

### Study characteristics

The 19 studies included in this systematic review involved a total of 643,093 participants, of which 54% were male [21–39]. Ten studies included both male and female participants [21, 25–28, 30, 32, 33, 37, 39], six studies included only male participants [22, 23, 29, 35, 36, 38], two studies included only female participants [24, 34], and one study did not report on the binary sex of their participants [31]. The age of participants ranged from 15 years [27] to 88 years [33]. Out of 19 studies, six studies originated in the United States [24–26, 29, 36, 39], four studies each in Canada [21, 30, 33, 37] and the United Kingdom [27, 28, 34, 35], and one study each in Australia [23], Finland [31], Germany [32], Italy [22], and China [38]. Nine of the 19 studies were cohort studies [21, 24, 26–30, 33, 39], one was a randomized controlled trial [36], and the remaining nine were cross-sectional studies [22, 23, 25, 31, 32, 34, 35, 37, 38]. Detailed characteristics of the included studies can be found in Tables 1 and 2.

### Attributes of sex assessments

#### Testosterone levels

Four studies used calculated free testosterone (cFT) and total testosterone (TT) as biological attributes of sex [22, 24, 31, 36], of which one study also constructed a polygenic scores (PGS) for total testosterone and free testosterone using data from the UK Biobank and FinnGen [31]. Two studies used a chemiluminescence assay to measure TT [22, 31], and two studies measured TT using liquid chromatography-tandem mass spectrometry [24, 36]. cFT was calculated from serum albumin and sex hormone-binding globulin values [22, 24, 31] or by equilibrium dialysis [36]. TT values were reported in ng/dL [24, 36], ng/mL [22], and nmol/L [31]. cFT values were reported in pg/mL [22, 24], ng/dL [36], and nmol/L [31].

### Attributes of gender assessments

#### Masculinity, femininity, and androgyny traits

Eleven studies assessed masculinity, femininity, and androgyny using two measurement tools: the Bem Sex Role Inventory (BSRI) and the Personal Attributes Questionnaire (PAQ) [21, 25–28, 30, 32–34, 37, 39]. Eight studies used the BSRI to capture masculinity, femininity, and androgyny in male and female participants; five studies used the full-length version [25, 28, 33, 34, 37] and three studies used the BSRI-short form [21, 27, 30]. Three studies applied the PAQ to assess traits of masculinity in male and female participants [26, 32, 39]. Möller-Leimkühler and colleagues (2009) used the German Extended PAQ (GE-PAQ), a German version of the original tool [32].

#### Masculine gender roles and norms

Two studies captured measures of masculine gender roles using the standardized Masculine Gender Role Discrepancy Stress Scale (MGRDSS) [23, 38]. Two studies captured measures of masculine norms using the Conformity to Masculine Norms Inventory (CMNI) [29, 35]. Iwamoto and colleagues (2018) used the CMNI-29 items, a modified 29-item version of the original CMNI [29].

### Outcome assessment

Fourteen studies examined mental health outcomes, including depression [21, 22, 25, 29, 30, 32, 34–39], anxiety [21, 38], muscle dysmorphia [23], stress [21], suicidal ideation [27], and trauma symptoms [30]. Eight tools were used to measure depression: Beck Depression Inventory (BDI) [22, 29, 30, 39], Center for Epidemiological Studies-Depression (CES-D) Scale [25, 37, 38], Depression, Anxiety and Stress Scale (DASS-21) [21], Edinburgh Postnatal Depression Scale (EPDS) [35], Patient Health Questionnaire (PHQ-9) [36], Hospital Anxiety and Depression Scale (HADS) [34], WHO-5 Wellbeing Index [32], and Gotland Scale of Male Depression (GSMD) [32]. Two tools were used to measure anxiety: DASS-21 [21] and Self-Consciousness Scale (SCS) [38]. The DASS-21 was also used to measure stress [21]. The Muscle Dysmorphic Disorder Inventory (MDDI) was used to measure muscle dysmorphia [23], and the PTSD-Civilian Checklist was used to measure trauma symptoms [30].

Two studies investigated sexual health outcomes, including erectile function [22, 36], orgasmic function [22], and sexual function [36]. Erectile function and orgasmic function were measured using the International Index of Erectile Function (IIEF) Questionnaire [22, 36] and sexual function was measured using the Psychosexual Daily Questionnaire (PDQ) [36].

**Fig. 2:**
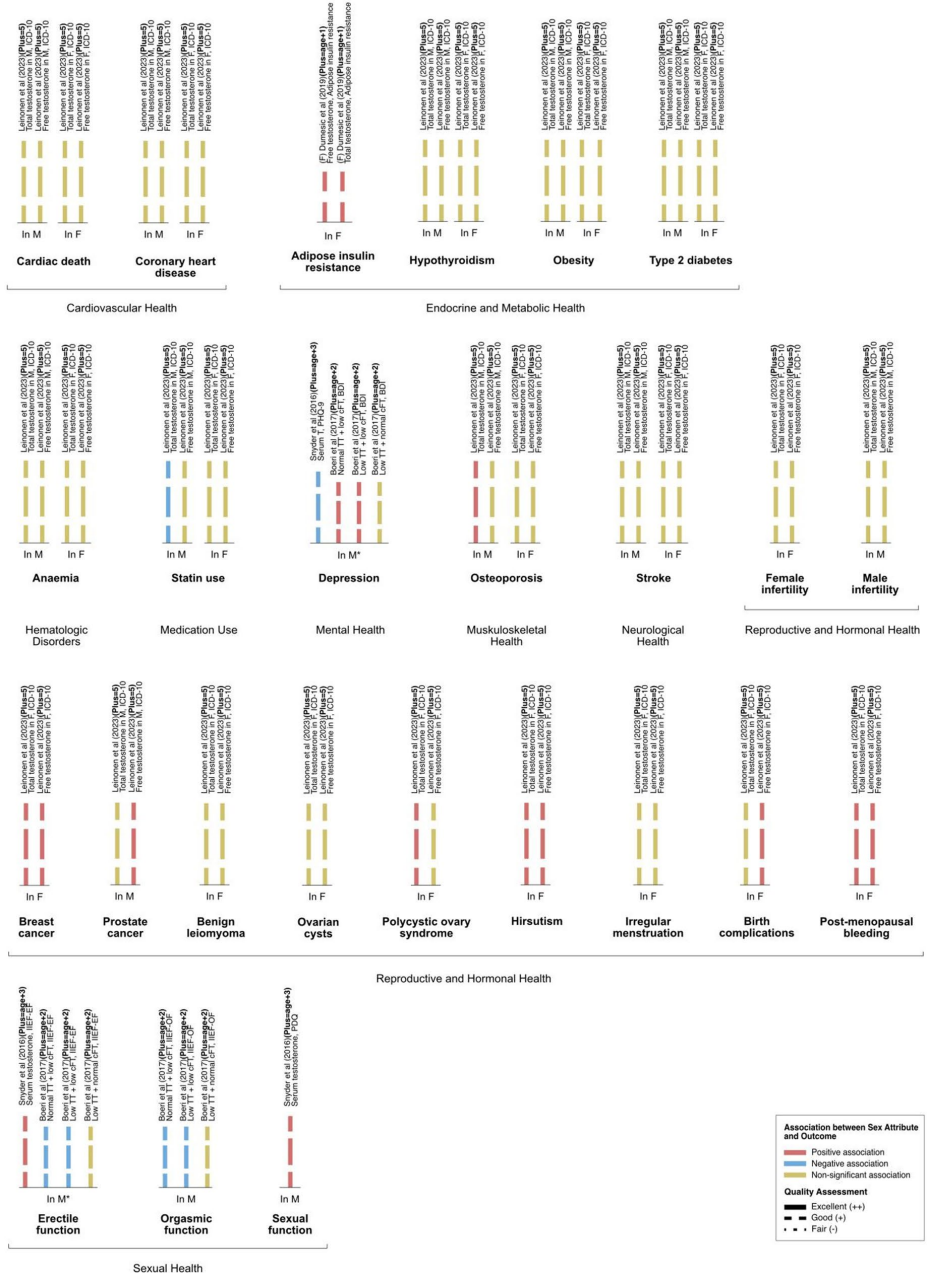
Associations between sex attribute (testosterone levels) and clinical outcome, organized by outcome. Color indicates direction of association between the sex attribute and clinical outcome: positive association (pink), negative association (blue), no statistically significant association (yellow). Bar labels indicate the author, number of PROGRESS-Plus variables controlled for in analysis, sex measure, outcome measure. Length of bars corresponds to the number of variables controlled for in analysis, categorized using the PROGRESS-Plus framework: P, place of residence; R, race; O, occupation; G, gender/sex; E, education; Ss, socioeconomic status; Sc, social capital; Plus, additional parameters. The number of Plus parameters is shown in parentheses; NR, not reported. Line style corresponds to Quality Assessment of the study: Excellent (++, solid lines), Good (+, dashed lines), Fair (-, dotted lines). Abbreviations: M, males; F, females

**Fig. 3:**
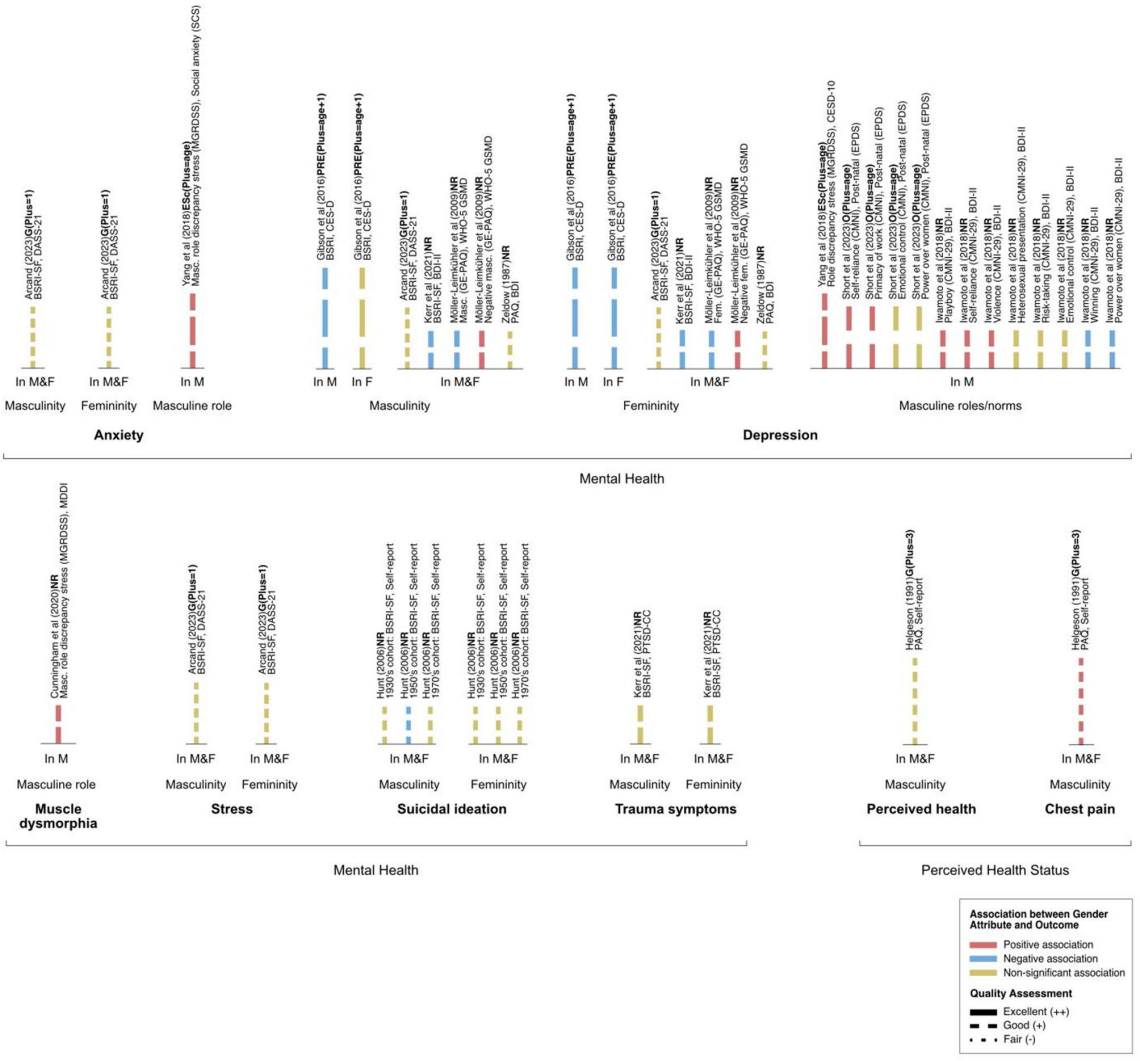
Associations between gender attributes and clinical outcome, organized by outcome. Color indicates direction of association between the gender attribute and clinical outcome: positive association (pink), negative association (blue), no statistically significant association (yellow). Bar labels indicate the author, number of PROGRESS-Plus variables controlled for in analysis, sex measure, outcome measure. Length of bars corresponds to the number of variables controlled for in analysis, categorized using the PROGRESS-Plus framework: P, place of residence; R, race; O, occupation; G, gender/sex; E, education; Ss, socioeconomic status; Sc, social capital; Plus, additional parameters. The number of Plus parameters is shown in parentheses; NR, not reported. Line style corresponds to Quality Assessment of the study: Excellent (++, solid lines), Good (+, dashed lines), Fair (-, dotted lines). Abbreviations: M, males; F, females

**Fig. 4:**
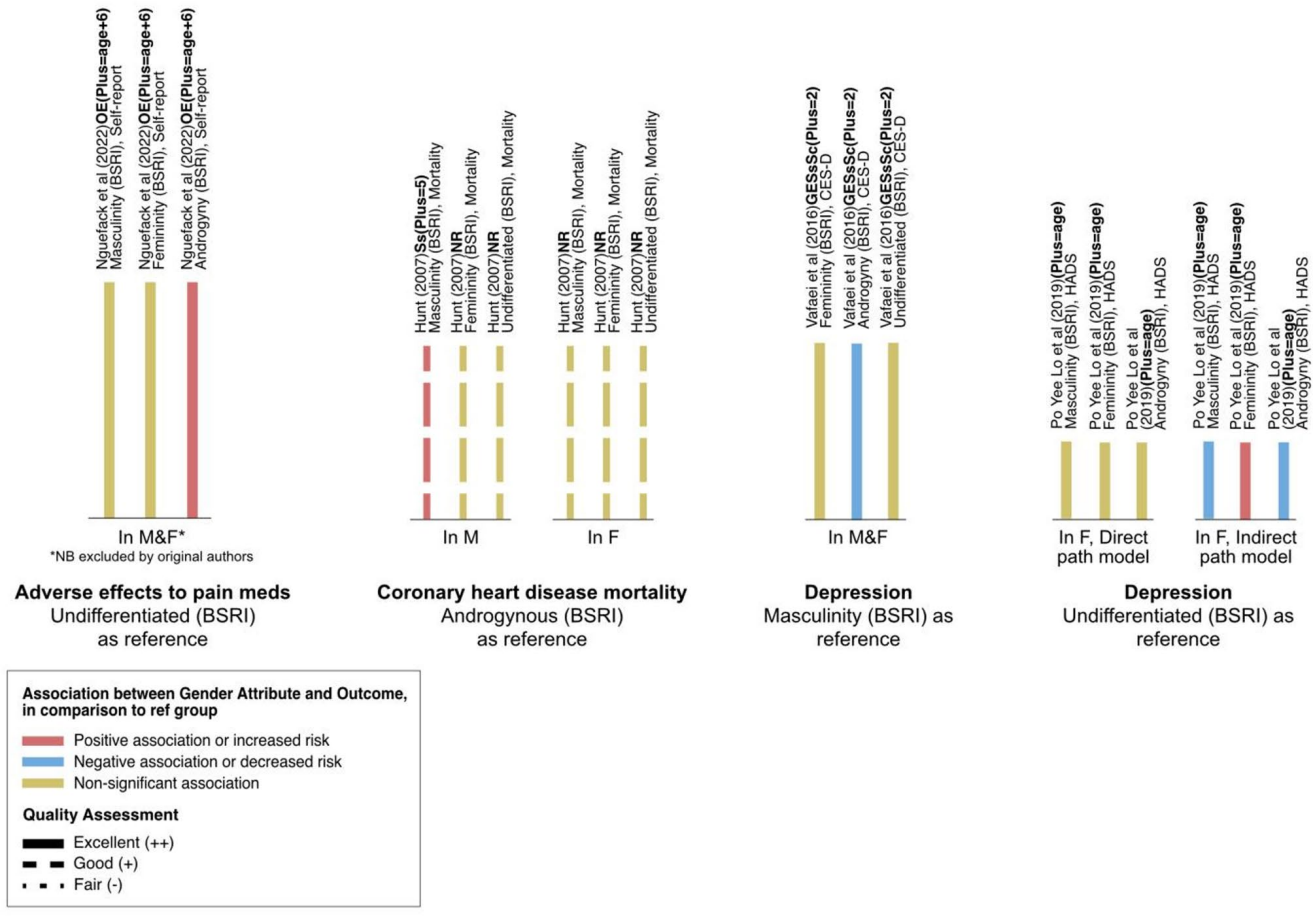
Associations between gender attributes and clinical outcome in comparison to a reference group, organized by outcome. Color indicates direction of association between the gender attribute and clinical outcome: positive association or increased risk (pink), negative association or decreased risk (blue), no statistically significant association (yellow). Bar labels indicate the author, number of PROGRESS-Plus variables controlled for in analysis, sex measure, outcome measure. Length of bars corresponds to the number of variables controlled for in analysis, categorized using the PROGRESS-Plus framework: P, place of residence; R, race; O, occupation; G, gender/sex; E, education; Ss, socioeconomic status; Sc, social capital; Plus, additional parameters. The number of Plus parameters is shown in parentheses; NR, not reported. Line style corresponds to Quality Assessment of the study: Excellent (++, solid lines), Good (+, dashed lines), Fair (-, dotted lines). Abbreviations: M, males; F, females

Two studies examined cardiovascular health, with outcomes of coronary heart disease (CHD) [31], cardiac death [31], and mortality from CHD [28]. Two studies investigated endocrine and metabolic disorders, including adipose insulin resistance [24], hypothyroidism, obesity, and type 2 diabetes [31]. One study examined self-reported side effects from chronic pain medications [33]. One study investigated perceived health status, including perceived health and self-reported chest pain post-myocardial infarction [26]. One of the included studies incorporated a large number of health outcomes into its analysis, including hematologic disorders (i.e., anaemia), medication use (i.e., statin use), musculoskeletal health (i.e., osteoporosis), neurological health (i.e., stroke), and reproductive and hormonal health (i.e., female and male infertility, breast cancer, prostate cancer, benign leiomyoma, ovary cysts, polycystic ovary syndrome (PCOS), hirsutism, irregular menstruation, birth complications, and postmenopausal bleeding) [31]. Associations between gender- and sex-related attributes and outcomes are summarized below, reported in Table 2, and illustrated in Figs. 2, 3 and 4.

### Relationship between sex attributes and outcomes

#### Cardiovascular health

Leinonen and colleagues (2023) reported non-significant associations between TT and free testosterone with cardiac death and with CHD in both males and females [31].

#### Endocrine and metabolic health

Leinonen and colleagues (2023) reported non-significant relationships between free testosterone and TT levels with hypothyroidism, obesity, and type 2 diabetes in both males and females [31]. Dumesic and colleagues (2019) found that in both groups of female participants (females with PCOS and age- and body mass index (BMI)-matched controls), cFT and TT were both positively correlated with adipose-insulin resistance [24].

#### Hematologic disorders

Leinonen and colleagues (2023) reported non-significant associations between free testosterone and TT levels with anaemia [31].

#### Medication use

Leinonen and colleagues (2023) found that in male participants, TT had a negative association with the use of statin and free testosterone had a non-significant association with statin use [31]. Findings in female participants were non-significant [31].

#### Mental health

Boeri and colleagues (2017) reported that participants with normal TT + low cFT and participants with low TT + low cFT had an increased occurrence of depression; they also reported that in participants with low TT + normal cFT, there was a non-significant association with depression [22]. Researchers concluded that low cFT levels independently predicted increased depression regardless of TT level, and that TT level had a non-significant association with depression [22]. Snyder and colleagues (2016) reported that in the treatment group, when the serum testosterone level was increased to within normal range for males aged 19–40 years, there was a decrease in depressive symptoms based on measurements captured by the PHQ-9 scale [36].

#### Musculoskeletal health

Leinonen and colleagues (2023) reported that TT in male participants had a positive association with osteoporosis [31]. All other associations were non-significant [31].

#### Neurological health

Leinonen and colleagues (2023) found non-significant associations between TT and free testosterone with stroke in both male and female participants.

#### Reproductive and hormonal health

Leinonen and colleagues (2023) investigated a number of outcomes related to reproductive and hormonal health [31]. The authors reported positive associations between both TT and free testosterone with breast cancer, hirsutism, and post-menopausal bleeding in female participants. The authors reported a positive association between TT and PCOS in females; the association with free testosterone was non-significant. Free testosterone was positively associated with prostate cancer in males and with birth complications in females; the association with TT was non-significant. The authors reported non-significant associations between TT and free testosterone with female infertility, benign leiomyoma, ovarian cysts, and irregular menstruation in female participants, and with male infertility in male participants.

#### Sexual health

Boeri and colleagues (2017) reported that participants with normal TT + low cFT and participants with low TT + low cFT had decreased erectile function and decreased orgasmic function [22]. Researchers also reported that in participants with low TT + normal cFT, there was a non-significant association with erectile function and orgasmic function, and concluded that low cFT, regardless of TT level, independently predicted decrease erectile and orgasmic function, measured with the IIEF [22]. Snyder and colleagues (2016) reported that when the serum testosterone level was increased to within normal range for males aged 19–40 years, there was a positive impact on erectile function and sexual function as measured by the IIEF and PDQ, respectively [36].

### Relationship between gender attributes and outcomes

#### Mental health

##### Anxiety

In their sample of male and female participants, Arcand and colleagues (2023) reported non-significant associations between femininity and masculinity scores with anxiety, as measured by the DASS-21 tool [21].

##### Depression

Gibson and colleagues (2016) reported that both high masculinity and high femininity were associated with decreased depression in male participants [25]. In female participants, high masculinity was not significantly associated with depression, but high femininity was associated with decreased depression, as measured with the CES-D Scale [25]. In their sample of male and female participants, Arcand and colleagues (2023) reported that both femininity and masculinity had non-significant associations with depression, as scored by the DASS-21 [21]. Möller-Leimkühler and colleagues (2009) reported that both masculinity and femininity were associated with decreased depression scores, and that ‘negative masculinity’ and ‘negative femininity’, as defined and measured by the GE-PAQ, were positively associated with depression [32]. Kerr and colleagues (2021) reported that both masculinity and femininity were associated with decreased depression scores in their sample of male and female participants [30]. Zeldow and colleagues (1987) reported that both masculinity and femininity had non-significant associations with depression [39]. Both teams of researchers measured depression scores with the BDI [30, 39].

##### Stress

In their sample of male and female participants, Arcand and colleagues (2023) reported non-significant associations between femininity and masculinity scores with stress, as measured by the DASS-21 [21].

##### Suicidal ideation

Hunt and colleagues (2006) reported that in the group of male and female participants born in the 1950 s (i.e., 1950 s cohort), masculinity had a negative association with self-reported suicidal ideation; the association was non-significant for the 1930 s and 1970 s cohorts [27]. Femininity had a non-significant association with self-reported suicidal ideation in all three cohorts [27].

##### Trauma symptoms

Kerr and colleagues (2021) reported that in a population of psychiatric hospital employees, there was no significant association between masculinity and femininity with trauma symptoms, as measured by the PTSD-CC [30].

#### Perceived health

Helgeson (1991) reported that masculinity had a non-significant association with self-reported perceived health in the sample of male and female participants post-myocardial infarction [26]. The authors reported that masculinity was positively associated with self-reported chest pain post-myocardial infarction [26].

### Relationship between masculine gender roles & norms and outcomes

#### Mental health

##### Anxiety

Yang and colleagues (2018) reported that in males, increased masculine role discrepancy stress was associated with increased social anxiety, which was measured using the SCS [38].

##### Depression

Yang and colleagues (2018) reported that in males, increased masculine role discrepancy stress was associated with increased depression, as measured by the CES-D [38]. Iwamoto and colleagues (2018) found that in males who had higher adherence to the masculine norms of “playboy”, “self-reliance”, and “violence”, there was an increase in depression scores, and in males who had higher adherence to the masculine norms of “winning” and “power over women”, there was a decrease in depression scores as scored by the BDI-II [29]. Non-significant associations were reported for norms of “heterosexual presentation”, “risk-taking”, and “emotional control” [29]. Short and colleagues (2023) reported that in males, adherence to the masculine norms of “self-reliance” and “primacy of work” was correlated with an increase in post-natal depression among fathers, measured using the EPDS [35]. Non-significant associations were reported for norms of “emotional control” and “power over women” [35].

##### Muscle dysmorphia

Cunningham and colleagues (2020) reported that in males, increased masculine discrepancy stress was associated with increased muscle dysmorphia, as scored by the MDDI [23].

### Relationship between gender attributes and outcomes with interaction terms

Three studies incorporated interaction terms in their analyses, encompassing gender identity and other parameters [21, 25, 33]. The results of these studies are described below and were not used in the certainty assessment.

#### Anxiety, stress, and depression

Arcand and colleagues (2023) studied the interactions between binary sex (i.e., male or female), gender attributes (i.e., femininity and masculinity), and time elapsed since baseline (i.e., T_1_ = 3 months, T_2_ = 6 months, T_3_ = 9 months, T_4_ = 12 months) and how these interactions associated with depression, anxiety, and stress [21]. Researchers identified a time*sex*femininity interaction for anxiety and stress, suggesting that female participants with low femininity levels had significantly higher anxiety symptoms than male participants with low femininity levels at T_4_, and that female participants with high femininity levels had significantly higher stress symptoms than male participants with high femininity at T_1_ [21]. There was no significant interaction detected between these parameters for depression [21]. Gibson and colleagues (2016) investigated the interaction between binary sex (i.e., male and female), gender attributes (i.e., masculinity and femininity), and education level (i.e., college educated and non-college educated) [25]. The results of this analysis indicated a significant interaction between masculinity and education, and suggested that increased masculinity was associated with an increase in depressive symptoms in college educated female participants, and associated with a decrease in depressive symptoms in non-college educated female participants [25]. The analysis also identified that among college educated male participants, increased femininity was associated with a decrease in depression levels compared to non-college educated male participants [25]. All other interactions were found to be not significant [25].

#### Adverse events from pain medication

Nguefack and colleagues (2022) studied the interaction between participants’ gender identity (i.e., man or woman) and their gender attributes (i.e., masculinity, femininity, or androgyny), and how these parameters associated with self-reported adverse effects from pain medication [33]. Results indicated that in participants who are classified as masculine and participants who are classified as androgynous, those who identify as men reported fewer severe adverse events as compared to those who identify as women [33].

### Relationship between gender identity and outcomes with reference groups

Four studies reported results in comparison to a reference group [28, 33, 34, 37]. Each of the four studies used the BSRI to capture participants’ masculinity, femininity, androgyny, and undifferentiated gender scores [28, 33, 34, 37]. Two studies used the undifferentiated group as a reference group in their analyses [33, 34], one study used the androgynous group as a reference group [28], and one study used masculinity as a reference group [37]. The results of these four studies are presented below and in Fig. 4. These results were not used in the certainty assessment.

#### Adverse events from pain medication

Nguefack and colleagues (2022) reported that androgyny (i.e., high masculinity *and* high femininity scores) was associated with a greater number of severe adverse effects to pain medication, in reference to participants with undifferentiated gender scores (i.e. low masculinity *and* low femininity scores) [33]. There was no significant association reported for participants with high masculinity nor high femininity scores [33]. This analysis was conducted in a sample of males and females; study authors excluded participants who self-identified as non-binary (*n* = 4) and justified this exclusion on the basis of statistical validity [33].

#### CHD mortality

Hunt and colleagues (2007) reported that high masculinity scores in male participants were associated with an increased risk of CHD mortality, in reference to males with high androgyny scores [28]. There was no significant increase or decrease in CHD mortality risk for males with high femininity nor undifferentiated scores, in reference to males with high androgyny scores [28]. There were no statistically significant results for the analysis conducted with female participants [28].

#### Depression

In their sample of male and female participants, Vafaei and colleagues (2016) reported that androgyny was associated with decreased prevalence rates of depression, in reference to participants with high masculinity scores [37]. There was no significant association between with high femininity nor undifferentiated scores with prevalence rates of depression, in reference to participants with high masculinity scores [37]. In their sample of female participants, Po Yee Lo and colleagues (2019) reported that masculine and androgynous traits were associated with decreased depression scores, in reference to females with undifferentiated gender scores; meanwhile, feminine traits were associated with increased depression scores [34].

### Sensitivity analysis

The results of sensitivity analysis suggest that study quality may explain the variability in the direction of the associations reported. This analysis was only possible for outcomes with results from at least two studies of different study quality.

Two studies of good quality [30, 32] and two studies of fair quality [21, 39] showed distinct results on the association between masculinity and depression. The two studies of good quality reported that higher levels of masculinity, captured using BSRI and GE-PAQ, are negatively associated with depression [30, 32]. Möller-Leimkühler and colleagues also reported a positive association between negative masculinity, as measured by the GE-PAQ and defined as undesirable attributes such as “egoistical” and “cynical”, and depression [32]. The two studies of fair quality reported no significant association between masculinity, captured using BSRI-SF and PAQ, and depression [21, 39].

The same four studies also investigated the association between femininity and depression. The direction of association was identical to those reported for masculinity. The two studies of good quality reported that higher levels of femininity are negatively associated with depression [30, 32]; negative femininity, measured with two subscales “verbal passive-aggressiveness” and “excessive selflessness”, was positively associated with depression [32]. The two studies of fair quality reported no significant association between femininity and depression [21, 39].

### Risk of bias and certainty of evidence

We used the QUIPS tool to rate the studies as “excellent” (i.e., low risk of bias), “good” (i.e., moderate risk of bias, or “fair” (i.e., high risk of bias) [18]. The six potential sources for bias were scored as “Yes”, “No”, “Cannot determine”, “Not applicable”, or “Not reported”. We rated three studies as “excellent” [33, 34, 37], twelve studies as “good” [22–25, 28–32, 35, 36, 38], and four as “fair” quality [21, 26, 27, 39] (Supplementary Material S5). We documented all sources of disagreement and decisions made (Supplementary Material S6).

We observed consistent associations between testosterone level and depression, as well as testosterone and erectile function, reported by two studies of good quality [22, 36]. Snyder et al. (2016) reported that increase in serum testosterone was negatively associated with depression [36] and Boeri et al. (2017) reported that low cFT was associated with increased depression [22]. For erectile function, Snyder et al. (2016) reported a positive association with serum testosterone [36] and Boeri et al. (2017) reported that low cFT was associated with a decrease in erectile function [22]. We considered these results to be the only ones indicative of evidence of moderate certainty.

We regarded all other evidence as very low in certainty due to heterogeneity in the direction and significance of the associations between sex and gender attributes and clinical outcomes.

### Missing data

We did not identify any missing or unclear data and thus it was not necessary to contact the primary authors of any of our included studies.

## Discussion

In our systematic review, we synthesized scientific evidence about the relationship between sex and gender attributes and clinically relevant outcomes with the goal of identifying a set of biological sex and sociocultural gender attributes that are important for clinical and research consideration. All 19 included studies assessed the relevance of sex and gender attributes (e.g., hormone level, gender identity, and gender norms) for clinical outcomes, however, the consistency, direction, and precision of associations differed greatly. This was not unexpected given the heterogeneity observed in characteristics of research participants, types of measures used to capture sex and gender attributes, measures of outcomes, type and number of controlling variables, and statistical approaches to study associations.

Testosterone level, a key hormone in reproductive and sexual health, emerged in our review as a consistent and important attribute with differing implications for the sexes [6]. Leinonen and colleagues (2023) reported positive associations between both TT and free testosterone with breast cancer, hirsutism, and post-menopausal bleeding in female participants [31]. In males, free testosterone was positively associated with prostate cancer [31]. Although these associations were robust after adjustment for a number of controlling variables, the authors suggested that future research should consider bidirectional associations between testosterone levels and the outcomes separately in the sexes [31]. The study by Boeri and colleagues (2017) found that participants with normal TT but low cFT, as well as those with low values for both measures, experienced significant reductions in both erectile function and orgasmic function [22], bringing attention to bioavailable testosterone and methods to precisely capture testosterone levels in the evaluation and management of sexual dysfunction.

Results on cardiovascular health allow for discussion on the effect of sex attributes as well as gender. This is particularly important as the cardiovascular system is the body system most affected during the human lifespan and in response to the environment [40–42], manifesting in alterations in the heart rate, blood pressure, and baroreceptor physiologic control [43]. Sex differences in type and severity of arrhythmias, atherosclerosis, heart-rate variability, and disrupted sleep are well-documented [44, 45]. In one study included in this review, authors investigated the effect of sex, particularly through testosterone level, on cardiac death and CHD but found no significant association neither in male nor female participants [31]. Hunt and colleagues (2007) investigated the effect of gender identity, and reported that among male participants, higher levels of femininity were associated with a decreased risk of CHD mortality, but not in female participants or male participants with high masculinity traits [28]. They also reported that high masculinity in male participants was associated with an increased risk of CHD mortality, in reference to androgynous males [28]. Together, these results suggest that gender traits may affect cardiovascular health outcomes independently of biological sex through reactivity, coping behaviors, and interpersonal sensitivity [46], but further research that considers both sex and gender attributes concurrently is needed to test this hypothesis.

Results of our systematic review brought attention to the cost of non-adherence to traditional masculine norms and gender role expectations, particularly when male persons are not living up to the societal standards of masculinity in Western society [23, 29, 35, 38]. The results from Yang and colleagues (2018) that, in males, greater masculine role discrepancy stress was significantly associated with higher depressive symptoms [38], are consistent with the results from Iwamoto and colleagues (2018) who found that that males with higher adherence to the masculine norms of playboy, self-reliance, and violence exhibited increased depression scores [29]. Short and colleagues (2023) found that adherence to masculine norms of self-reliance and primacy of work associate with higher postnatal depression among new fathers [35], provoking discussion on the internalized distress of prioritizing work above family needs. Combined with the results of Iwamoto and colleagues (2018), that adherence to the masculine norms of winning and power over women was associated with decreased depression scores [29], and that of Yang and colleagues (2018) who found that males experiencing higher levels of masculine role discrepancy stress reported increased social anxiety [38], we highlight the complex and conflicting dimensions played by gender in the relationships between the sexes, and the conflict between societal expectations and personal identity on mental health [47, 48]. At the same time, the role of sex attributes in mental health should not be overlooked. Findings from Snyder and colleagues (2016) and Boeri and colleagues (2017) point to the association between testosterone levels in males and depression scores [22, 36]. Taken together, these results emphasize that the processes underlying health and disease are complex, possibly shaped by a continuous interplay of biological sex through genetic, hormonal, and physiological factors, as well as sociocultural gender through roles, responsibilities, and relationships [7, 49].

An important point that warrants attention is the state of evidence regarding feminine gender norms and role expectations in relation to clinically relevant outcomes. While several studies examined masculine attributes, norms, and behaviors [23, 29, 35, 38], there remains a significant gap in research addressing the effect of feminine roles and expectations on clinically relevant outcomes. This omission is striking, as societal norms surrounding caregiving, household responsibilities, and emotional sensitivity are known to shape health behaviors and outcomes in ways distinct from masculine norms [50, 51]. The androcentric bias in the state of the evidence points to an avenue of future research that needs to address this imbalance.

### Study limitations

Heterogeneity across sample characteristics (e.g., age, sex and gender, and other factors), as well as definitions and measurements of outcomes precluded us from drawing definite conclusions, which was reflected in certainty assessment. While we observed variability in the reported associations and magnitude of effect sizes, evidence on the topic is extensive and provides a foundation on which to develop future research. The confounding influences of the wide age ranges of the samples requires special discussion. While we treated age as a key confounder in the quality assessment of the included studies (see Supplement S5, item 14), given its well-documented effects on sex steroid hormone levels and gendered role expectations, study participants’ age ranges spanned many years. We emphasize that future studies should investigate sex and gender in clinically relevant outcomes with greater precision in age, as sex-specific physiology and gender-specific norms and role expectations are often age-dependent. The integration of advanced statistical techniques and machine learning could lead to a more precise identification of a critical age period in the study samples [52], should they exist, which is hardly feasible through traditional hypothesis-driven analytic methodologies that assume linearity in the relationship between variables.

In line with our protocol, we included all observational studies on the topic that used standardized measures applied by at least two different research teams, aiming to enhance comparability across studies and findings. However, many studies meeting the inclusion criteria did not apply the same measures, and the characteristics of research participants of the study samples varied greatly. This led to the exclusion of potentially relevant measures and outcomes from our review, since they were applied by only one research team.

We limited our search to studies published in English, which may affect the generalizability of our findings due to the omission of papers published in other languages. Despite these limitations, our review is the first comprehensive assessment of the effects of sex and gender, measured using standardized tools, on clinically relevant outcomes.

### Clinical implications

Our systematic review presented a comprehensive synthesis of associations between sex and gender attributes and 34 clinically relevant outcomes across multiple domains, including cardiovascular health, mental health, endocrine and metabolic health, reproductive and hormonal health, sexual health, among others. The results of our systematic review raise several important clinical questions. Should patient management strategies be tailored by sex and gender? Is it possible that due to increased responsivity to steroid hormones, persons with mental health disorders may benefit more from psychological interventions, as opposed to pharmacotherapy? If future research addresses these important clinical questions, incorporating both sex and gender attributes concurrently into the clinical assessment of disorders of metabolism and reproduction, mental health conditions, and other related issues may well become standard practice.

### Research implications

In our systematic review, we synthesized published research that used standardized measures of sex and gender attributes to study their implications in clinically relevant outcomes, as opposed to binary sex and gender. Of the 19 studies included in our review, none captured attributes of both sex and gender; each study measured either attributes of sex or gender, but not both. Incorporating standardized measurements of both sex and gender attributes in future health research may provide a richer understanding of the role that these constructs play in clinical outcomes.

We offer several recommendations to guide future research. In alignment with guidance from various health and research authorities, we emphasize incorporation of standardized measures of both biological sex and sociocultural gender attributes in research with human participants. The results would allow investigators to identify sex- and gender-specific targets, thereby seeding the pipeline with the potential to produce precision interventions. This line of research is gaining attention, particularly using big data to build gender indexes taking into account biological sex and apply them to the investigation of risks for clinical outcomes [49, 53, 54]. Second, future research should focus on elucidating biopsychosocial mechanisms driving differences in clinically relevant outcomes [55], in lieu of additional descriptive studies that demonstrate yet again that people are diverse in both their biology as well as their gender. This recommendation will be best served by translational approaches that take novel findings from research across health pillars, as this is a critical tenet of the biopsychosocial model that is often overlooked. Third, research should routinely include description and analyses of sex and gender attributes of their research participants, even if these findings are presented in supplementary tables. Very limited information regarding such characteristics is available in published research. Building on the existing research base by following the above recommendations holds the potential to substantively impact evidence-based care in the foreseeable future.

We also propose several methodological recommendations for future research. Studies examining the effect of sex and gender attributes on various clinically relevant outcomes require focus on methodologies that can capture these effects. Several studies included in this review adjusted their sex attribute-related results for age. Likewise, several studies included in this review adjusted their gender attribute-related results for binary sex and age. These studies were primarily motivated to use and select these covariates for statistical adjustment to increase the study’s internal validity by correcting the data and eliminating confounding effect. However, no adjustments should be undertaken until subgroup-specific results have been studied [56]. The pooling of data across sexes and ages not only assumed no difference between sexes and ages but also prevented the researchers from testing an outcome’s independency on a participant’s sex and/or age [57, 58]. Several studies combined the sexes in one group in statistical analyses to increase the study power. However, this approach results in bias due to uneven size of each sex in the study sample. Female participants were underrepresented in most studies, and this can alter clinically relevant outcome scores. Systemic barriers may limit females’ participation in research due to childbearing and gender norms and role expectations. Having both variances and unequal sample sizes in the study cohort creates difficulty in accounting for confounding variables.

Finally, neuroscience and behavioral sciences challenge the binary view of sex and gender and call for researchers to take a more inclusive approach to better understand sex and gender attributes, and how they relate to clinically relevant outcomes [59, 60]. However, analytical methods that would allow for defining samples based on sex and gender variability are yet to be developed, and are expected to arrive with the emergence of personalized medicine and precision public health.

## Conclusions

Results from the studies we reviewed suggest that neither sex nor gender attributes should be overlooked when investigating clinically relevant outcomes. Ignoring these associations may confound interpretation of results and mask the true effects that could be subject to modification to improve outcomes. Investigating sex and gender attributes concurrently might also contribute to a better understanding of the differences that are observed in disorders and diseases that disproportionately affect one sex more than others. Researchers are becoming more aware of the interactive nature of biological variables and the sociocultural environment, and their combined influence on gender identity. Such interactions are particularly important to consider when interpreting differences in the associations reported in the research included in this review, which solely focused on either sex or gender.

## Supplementary Information

Supplementary Material 1.

Supplementary Material 2.

Supplementary Material 3.

Supplementary Material 4.

Supplementary Material 5.

Supplementary Material 6.

Supplementary Material 7.

## Data Availability

The data and materials supporting the findings of this systematic review are available from the corresponding author upon reasonable request.

## Abbreviations

BDI: Beck depression inventory
BMI: Body mass index
BSRI: Bem sex role inventory
CADTH: Canadian agency for drugs and technologies in health
CES-D: Center for epidemiological studies-depression
cFT: Calculated free testosterone
CHD: Coronary heart disease
CIHR: Canadian institutes of health research
CMNI: Conformity to masculine norms inventory
DASS-21: Depression, anxiety and stress scale
EPDS: Edinburgh postnatal depression scale
GE-PAQ: German extended personal attributes questionnaire
GRADE: Grading of recommendations assessment, development and evaluation
GSMD: Gotland scale of male depression
HADS: Hospital anxiety and depression scale
IIEF: International index of erectile function
MDDI: Muscle dysmorphic disorder inventory
MGRDSS: Masculine gender role discrepancy stress scale
PAQ: Personal attributes questionnaire
PCOS: Polycystic ovary syndrome
PDQ: Psychosexual daily questionnaire
PHQ-9: Patient health questionnaire
PRISMA: Preferred reporting items for systematic reviews and meta-analyses
PTSD-CC: Post traumatic stress disorder-civilian checklist
PTSD: Post-traumatic stress disorder
QUIPS: Quality in prognosis studies
SCS: Self-consciousness scale
TT: Total testosterone
WHO-5: World health organization wellbeing index
