# Supplementary material for: The effects of sex and gender attributes on clinical outcomes: a systematic review
Source: Biol Sex Differ. 2025 Dec 29;16:108. doi: 10.1186/s13293-025-00772-x (PMC12751436; doi:10.1186/s13293-025-00772-x)
Supplement: Supplementary file 2 — Supplementary Material 2. [file 13293_2025_772_MOESM2_ESM.pdf]

NIHR | National Institute for Health and Care Research

PROSPERO
International prospective register of systematic reviews

Instruments measuring sex and gender in biomedical research: A systematic review

Tatyana Mollayeva, Emilia Main

To enable PROSPERO to focus on COVID-19 submissions, this registration record has undergone basic automated checks for eligibility and is published exactly as submitted. PROSPERO has never provided peer review, and usual checking by the PROSPERO team does not endorse content. Therefore, automatically published records should be treated as any other PROSPERO registration. Further detail is provided here.

Citation
Tatyana Mollayeva, Emilia Main. Instruments measuring sex and gender in biomedical research: A systematic review. PROSPERO 2024 Available from https://www.crd.york.ac.uk/PROSPERO/view/CRD42023456917

REVIEW TITLE AND BASIC DETAILS

Review title
Instruments measuring sex and gender in biomedical research: A systematic review

Original language title
English

Review objectives

The objectives of the systematic review are to: (1) describe each instrument's key measurement properties (i.e., construct validity); (2) classify instruments according to the sex and gender markers they assess; and (3) summarize information relevant to their clinical and research applications.

P: Clinical or general population samples or population-based data

I: Not applicable

C: Sex and/versus gender

O: Any clinical or functional outcome

S: Observational studies

Keywords

attitudes behaviours, femininity, gender measure, gender role norms, gender stereotypes, masculinity, sex measure, sex/gender sensitivity, steroid hormones

SEARCHING AND SCREENING

Searches

Search terms used (subject to be edited/modified):

gender OR gender measure* OR sex/gender sensitivity OR gender role norms

attitudes behaviours OR gender stereotypes OR gender stereotyping OR social-construct OR socially-constructed AND (masculine* OR feminin* OR male OR female) OR masculinities OR femininities OR gender practice OR conformity to masculine norms OR conformity to feminine norms AND (Measurement OR Measuring OR measure* OR personality inventory OR instrument*

Study design

Cross-sectional for assessment (screening) and cohort for evaluation (prognosis)- subject to be refined

ELIGIBILITY CRITERIA

Condition or domain being studied

Condition #1. It is important to recognize that, depending on the people involved in research, people may identify with multiple sexes, genders, and orientations simultaneously. Depending on the purpose of the assessment or evaluation, the measure may need to consider some or all of these dimensions simultaneously. Unfortunately, we lack adequate information regarding the development and psychometric properties of many existing instruments designed to measure biological sex and gender. This

includes the method(s) by which the measure/instrument was developed, the reliability and validity of the measure, and the populations in which it has been used to predict clinical and functional outcomes of neurological disorders and injuries. As such, this research will report on measures/instruments that have theit process of development explicitly reported and published.

Population

Clinical or general population samples or population-based data

Intervention(s) or exposure(s)

Not applicable

Comparator(s) or control(s)

Sex versus gender

Context

Studies to date have used numerous measures of sex and gender, studying differences in clinical and functional outcomes because of differences between the sexes in biology/physiology (sex differences) and differences in roles, responsibilities, relationships and identities (i.e., gender differences) The results research produced so far, even within the same clinical population (i.e., traumatic brain injury) were inconsistent, even when stratifying results by time since injury and injury severity. To elucidate the source of these inconsistencies, it is important to examine the measures of sex and gender that have been utilized in research and to assess their ability to capture sex and/or gender. Instruments used to capture a construct are termed "descriptive" and those that capture change over time are termed ‘evaluative’. Measuring sex and gender is challenging because there is no generally accepted reference or gold standard instrument that accurately defines and measures the constructs, against which all new instruments could be compared (convergent validity, a subcategory of construct validity). Divergent validity is another subcategory of construct validity, and it involves the assessment of the discrepancy between constructs believed to be different (e.g., sex and gender) and thus expected to yield scores on their respective measures that are not strongly correlated. Another subcategory of construct validity is known-groups validity, which refers to the application of an instrument to two groups known or hypothesized to differ in the construct measured. The goal is to identify instrument(s) for measuring sex and gender todate in biomedical research.

OUTCOMES TO BE ANALYSED

Main outcomes

Any clinical or functional outcome (main focus outcomes concerning brain health)

Measures of effect

Relative risks, odds ratios, risk difference or any other measure used by researchers to discuss sex and/or gender effects

Additional outcomes

None

Measures of effect

Not applicable

DATA COLLECTION PROCESS

Data extraction (selection and coding)

Descriptive aspects of each instrument: (i) general: purpose, content, response options, (ii) application: how to obtain, method of scoring and interpretation; (iii) critical appraisal: strengths, considerations, applicability; (iv) markers (biological, behavioural, or social) it captures

Risk of bias (quality) assessment

Previously developed standardized forms developed for our prior research on measurements will be used to assess study quality and to synthesize results. Study quality will be assessed using the Quality in Prognosis Studies (QUIPS) guidelines. Assessments will be based on the presence of six potential sources of bias (i.e., participation, attrition, prognostic factors, outcome measurements, confounding measurements and account, and data analyses). Each study will be assigned an overall “risk of bias”, and those with the greatest risk were excluded. Studies of a retrospective nature will be automatically excluded from a “low risk” rating, as recommended by the Scottish Intercollegiate Guidelines Network (SIGN) criteria.

PLANNED DATA SYNTHESIS

Strategy for data synthesis

Criteria for evidence-based assessment proposed by Holmbeck and colleagues will be utilized. Instruments used at least twice in studies identified in the primary search will be given ratings of “well-established”, “approaching well-established” or “promising”, based on the following criteria: (1) use in peer-reviewed studies by different research teams; (2) availability of sufficient information for critical appraisal and replication; and (3) demonstration of validity and reliability in the studied population.

Analysis of subgroups or subsets

Categorization of instruments measuring sex and gender by content

Descriptive aspects of instruments measuring sex and gender

REVIEW AFFILIATION, FUNDING AND PEER REVIEW

Review team members

- Dr Tatyana Mollayeva, Toronto Rehabilitation Institute
- Ms Emilia Main, University Health Network

Review affiliation

University Health Network / University of Toronto

Funding source

Canada Research Chair in Neurological Disorders and Brain Health (CRC-2021-00074) and

Global Brain Health Institute (GBHI), Alzheimer\u2019s Association, and the Alzheimer\u2019s Society UK Pilot Awards for Global Brain Health Leaders (GBHI ALZ UK-23-971123)<\/div>

Named contact

Tatyana Mollayeva. 550 University Ave\\ Toronto, ON M5G 2A2 Canada
tatyana.mollayeva@utoronto.ca

TIMELINE OF THE REVIEW

Review timeline

Start date: 01 August 2023. End date: 31 December 2024

Date of first submission to PROSPERO

23 August 2023

Date of registration in PROSPERO

02 September 2023

CURRENT REVIEW STAGE

Publication of review results

The intention is to publish the review once completed.The review will be published in English

Stage of the review at this submission

Review stage	Started	Completed
Pilot work	✓	
Formal searching/study identification	✓	
Screening search results against inclusion criteria		
Data extraction or receipt of IP		
Risk of bias/quality assessment		
Data synthesis		

Preliminary searches in MEDLINE

Review status

The review is currently planned or ongoing.

ADDITIONAL INFORMATION

Additional information

Collaborators

- **Ms Thaisa Tylinski Sant'Ana**, University of Toronto

PROSPERO version history

- Version 1.1 published on 02 Sep 2023
- Version 1.0 published on 02 Sep 2023

Review conflict of interest
None known

Country
Canada

Medical Subject Headings
Biomedical Research; Data Management; Female; Humans; Male; Outcome Assessment, Health Care

Details of any existing review of the same topic by the same authors
Mollayeva T, Mollayeva S, Pacheco N, Colantonio A. Systematic Review of Sex and Gender Effects in Traumatic Brain Injury: Equity in Clinical and Functional Outcomes. Front Neurol. 2021 Sep 10;12:678971. doi: 10.3389/fneur.2021.678971. PMID: 34566834; PMCID: PMC8461184.

Disclaimer
The content of this record displays the information provided by the review team. PROSPERO does not peer review registration records or endorse their content.

PROSPERO accepts and posts the information provided in good faith; responsibility for record content rests with the review team. The owner of this record has affirmed that the information provided is truthful and that they understand that deliberate provision of inaccurate information may be construed as scientific misconduct.

PROSPERO does not accept any liability for the content provided in this record or for its use. Readers use the information provided in this record at their own risk.

Any enquiries about the record should be referred to the named review contact
