# Supplementary material for: The effects of sex and gender attributes on clinical outcomes: a systematic review
Source: Biol Sex Differ. 2025 Dec 29;16:108. doi: 10.1186/s13293-025-00772-x (PMC12751436; doi:10.1186/s13293-025-00772-x)
Supplement: Supplementary file 3 — Supplementary Material 3. [file 13293_2025_772_MOESM3_ESM.docx]

**Supplement 3. Search Strategy for one database**

**Database:** Ovid MEDLINE(R) ALL <1946 to November 17, 2023>

| **#** | **Query** | **Results from 20 Nov 2023** |
| --- | --- | --- |
| 1 | exp Gender Identity/ or exp "Sexual and Gender Minorities"/ | 38,918 |
| 2 | Gender Dysphoria/ or Transvestism/ | 1,583 |
| 3 | ((gender identit* or sex role* or masculin* or feminin* or effemina* or androgyn* or transgender* or transsexual* or transvest* or cross dress* or crossdress* or non binary or nonbinary) adj5 (survey* or questionnaire* or instrument* or form or forms or measur* or self report* or tool* or test* or indices or scale* or inventor* or checklist* or check list* or item* or score* or scoring or outcome* or data* or information or statistic*)).ti,ab,kf. | 5,014 |
| 4 | ((gender* or sex or sexes or male* or female* or man or men or woman or women) adj2 (role* or attitude* or percept* or sensitiv* or difference* or characteristic* or factor* or based or related or conform* or nonconform* or norm or norms or stereotyp* or behavio?r* or perform* or present* or practic* or dysphor* or identit* or express* or binar* or fluid* or construct* or dimension* or effect* or experienc* or mechanism* or continuum or minorit* or divers*) adj2 (survey* or questionnaire* or instrument* or form or forms or measur* or self report* or tool* or test* or indices or scale* or inventor* or checklist* or check list* or item* or score* or scoring or outcome* or data* or information or statistic*)).ti,ab,kf. | 15,039 |
| 5 | ((gender* or sex role* or sex or sexes or masculin* or feminin* or effemina* or androgyn* or transgender* or transsexual* or transvest* or cross dress* or crossdress* or non binary or nonbinary) adj2 index*).ti,ab,kf. | 3,816 |
| 6 | ((gender* or sex or sexes or masculin* or feminin* or effemina* or androgyn* or transgender* or transsexual* or transvest* or cross dress* or crossdress* or non binary or nonbinary) adj2 (role* or attitude* or percept* or sensitiv* or conform* or nonconform* or norm or norms or stereotyp* or behavio?r* or perform* or present* or practic* or dysphor* or identit* or express* or binar* or fluid* or construct* or dimension* or effect* or experienc* or mechanism* or continuum or minorit* or divers*) adj2 (indicat* or assess* or study* or studied or explor* or identif* or examin* or evaluat* or determin* or investigat* or collect*)).ti,ab,kf. | 8,930 |
| 7 | or/1-6 [Gender] | 66,430 |
| 8 | exp Sexuality/ | 50,379 |
| 9 | ((sexualit* or sexual orientation* or sexual identit* or sexual preference* or heterosexual* or homosexual* or bisexual* or asexual* or psychosexual* or sociosexual*) adj5 (survey* or questionnaire* or instrument* or form or forms or measur* or self report* or tool* or test* or indices or scale* or inventor* or checklist* or check list* or item* or score* or scoring or outcome* or data* or information or statistic*)).ti,ab,kf. | 8,067 |
| 10 | ((sexualit* or sexual orientation* or sexual identit* or sexual preference* or heterosexual* or homosexual* or bisexual* or asexual* or psychosexual* or sociosexual*) adj2 index*).ti,ab,kf. | 41 |
| 11 | ((sexualit* or sexual orientation* or sexual identit* or sexual preference* or heterosexual* or homosexual* or bisexual* or asexual* or psychosexual* or sociosexual*) adj2 (indicat* or assess* or study* or studied or explor* or identif* or examin* or evaluat* or determin* or investigat* or collect*)).ti,ab,kf. | 4,201 |
| 12 | or/8-11 [Sexuality] | 56,509 |
| 13 | ("Surveys and Questionnaires"/ or Checklist/ or Self Report/ or Personality Assessment/ or Personality tests/ or Personality inventory/ or Psychological Tests/) and (valid* or reliability or reliable or reproducib* or property or properties or factor structure or factor analysis or item analysis or unreliable or invalid* or error* or invariance).ti,ab,kf. | 127,869 |
| 14 | (exp "Reproducibility of Results"/ or exp Factor Analysis, Statistical/ or Validation Study/) and (survey* or questionnaire* or instrument* or form or forms or measure or measures or self report* or tool* or test or tests or index or indexes or indices or scale* or inventor* or checklist* or check list* or item* or score* or scoring).ti,ab,kf. | 318,213 |
| 15 | ("Surveys and Questionnaires"/ or Checklist/ or Self Report/ or Personality Assessment/ or personality tests/ or personality inventory/ or Psychological Tests/) and (exp "Reproducibility of Results"/ or exp Factor Analysis, Statistical/ or Validation Study/) | 72,909 |
| 16 | ((survey* or questionnaire* or instrument* or form or forms or measure or measures or self report* or tool* or test or tests or index or indexes or indices or scale* or inventor* or checklist* or check list* or item* or score* or scoring) adj3 (valid* or reliability or reliable or reproducib* or property or properties or factor structure or factor analysis or item analysis or unreliable or invalid* or error* or invariance)).ti,ab,kf. | 269,079 |
| 17 | ((survey* or questionnaire* or instrument* or form or forms or measure or measures or self report* or tool* or test or tests or index or indexes or indices or scale* or inventor* or checklist* or check list* or item* or score* or scoring) adj3 (creat* or develop* or design* or construct*)).ti,ab,kf. | 352,571 |
| 18 | ((survey* or questionnaire* or instrument* or form or forms or measure or measures or self report* or tool* or test or tests or index or indexes or indices or scale* or inventor* or checklist* or check list* or item* or score* or scoring) adj3 (interchangeab* or compar* or interscale or inter scale or agreement)).ti,ab,kf. | 219,403 |
| 19 | psychometrics/ or psychometric*.ti,ab,kf. | 117,650 |
| 20 | is.fs. | 688,788 |
| 21 | or/13-20 [Instruments] | 1,686,184 |
| 22 | (7 or 12) and 21 | 8,258 |
| 23 | ((gender* or sex role* or masculin* or feminin* or effemina* or androgyn* or transgender* or transsexual* or transvest* or cross dress* or crossdress* or non binary or nonbinary) adj5 measur*).ti. | 483 |
| 24 | ((gender* or sex role* or masculin* or feminin* or effemina* or androgyn* or transgender* or transsexual* or transvest* or cross dress* or crossdress* or non binary or nonbinary) adj3 measur*).ab,kf. | 3,293 |
| 25 | ((sexualit* or sexual orientation* or sexual identit* or sexual preference* or heterosexual* or homosexual* or bisexual* or asexual* or psychosexual* or sociosexual*) adj5 measur*).ti. | 103 |
| 26 | ((sexualit* or sexual orientation* or sexual identit* or sexual preference* or heterosexual* or homosexual* or bisexual* or asexual* or psychosexual* or sociosexual*) adj3 measur*).ab,kf. | 558 |
| 27 | or/23-26 | 4,203 |
| 28 | 22 or 27 | 11,908 |
| 29 | Epidemiologic Methods/ or exp Epidemiologic Studies/ or Observational Studies as Topic/ or Clinical Studies as Topic/ or Single-Case Studies as Topic/ | 3,235,990 |
| 30 | (Observational Study or Validation Study or Clinical Study).pt. | 262,235 |
| 31 | (observational adj3 (study or studies or design or analysis or analyses)).ti,ab,kf. | 230,890 |
| 32 | cohort*.ti,ab,kf. | 895,003 |
| 33 | (prospective adj7 (study or studies or design or analysis or analyses)).ti,ab,kf. | 547,242 |
| 34 | ((follow up or followup) adj7 (study or studies or design or analysis or analyses)).ti,ab,kf. | 173,935 |
| 35 | ((longitudinal or longterm or (long adj term)) adj7 (study or studies or design or analysis or analyses or data)).ti,ab,kf. | 356,732 |
| 36 | (retrospective adj7 (study or studies or design or analysis or analyses or data or review)).ti,ab,kf. | 708,681 |
| 37 | ((case adj control) or (case adj comparison) or (case adj controlled)).ti,ab,kf. | 163,286 |
| 38 | (case-referent adj3 (study or studies or design or analysis or analyses)).ti,ab,kf. | 640 |
| 39 | (population adj3 (study or studies or analysis or analyses)).ti,ab,kf. | 240,082 |
| 40 | (descriptive adj3 (study or studies or design or analysis or analyses)).ti,ab,kf. | 114,031 |
| 41 | ((multidimensional or (multi adj dimensional)) adj3 (study or studies or design or analysis or analyses)).ti,ab,kf. | 5,107 |
| 42 | (cross adj sectional adj7 (study or studies or design or research or analysis or analyses or survey or findings)).ti,ab,kf. | 456,214 |
| 43 | ((natural adj experiment) or (natural adj experiments)).ti,ab,kf. | 3,461 |
| 44 | (quasi adj (experiment or experiments or experimental)).ti,ab,kf. | 21,357 |
| 45 | ((non experiment or nonexperiment or non experimental or nonexperimental) adj3 (study or studies or design or analysis or analyses)).ti,ab,kf. | 1,760 |
| 46 | (prevalence adj3 (study or studies or analysis or analyses)).ti,ab,kf. | 51,131 |
| 47 | or/29-46 [CADTH [https://searchfilters.cadth.ca/link/38](https://urldefense.com/v3/__https:/searchfilters.cadth.ca/link/38__;!!CjcC7IQ!MOw7YyeCfVI-jSHqkJiwMYtZpRVdIh4X3KarFovH_h-lXxO9MUEDs68VU-5TN9jTlI8-1qccRWQvG0-QJjBJ$)] | 4,532,807 |
| 48 | 28 and 47 | 4,807 |
| 49 | 48 not ((child* or adolescen* or infan* or juvenile* or p?ediatric or p?ediatric) not (adult* or woman or man or women or men or middle age* or elder* or aged)).ti,ab,kf,hw. | 4,387 |
| 50 | (gender* or sex* or male* or female* or man or men or woman or women or masculin* or feminin* or effemina* or androgyn* or transgender* or transsexual* or transvest* or cross dress* or crossdress* or non binary or nonbinary or heterosexual* or homosexual* or bisexual* or asexual* or psychosexual* or sociosexual*).ti,kf. | 1,047,740 |
| 51 | (gender* or sex* or male* or female* or man or men or woman or women or masculin* or feminin* or effemina* or androgyn* or transgender* or transsexual* or transvest* or cross dress* or crossdress* or non binary or nonbinary or heterosexual* or homosexual* or bisexual* or asexual* or psychosexual* or sociosexual*).ab. /freq=2 | 2,030,492 |
| **52** | **49 and (1 or 2 or 8 or 50 or 51)** | **3,869** |

Filters

Medline

Observational Studies - MEDLINE. In: CADTH Search Filters Database. Ottawa: CADTH; 2023: https://searchfilters.cadth.ca/link/38. Accessed 2023-11-16.

Revisions:

- Removed Line 6 Case Reports as Topic/
- Removed lines 24-27 (case series.ti,ab,kf; case reports.pt; (case adj3 (report or reports or study or studies or histories)).ti,ab,kf; organizational case studies/)
- Revised line 7 (Validation Studies to Validation Study)
- Combined lines 1-5
