# Supplementary material for: The effects of sex and gender attributes on clinical outcomes: a systematic review
Source: Biol Sex Differ. 2025 Dec 29;16:108. doi: 10.1186/s13293-025-00772-x (PMC12751436; doi:10.1186/s13293-025-00772-x)
Supplement: Supplementary file 4 — Supplementary Material 4. [file 13293_2025_772_MOESM4_ESM.docx]

**Supplement 4**. Excluded studies and reason for exclusion

Reports not retrieved: Articles requested but could not be obtained

1. Alirezaei S, Azmoude E, Mokhtari N. Relationships between gender roles and attitudes towards menstruation and perception of menstrual pain in Iranian women. Current Women’s Health Reviews, 2021 June 11;13(3):2022.
2. Prunty SK. Differences in the symptoms of myocardial infarction by sex and gender identity. 1999;236.

| # | Citation | Reason for exclusion |
| --- | --- | --- |
|  | Abdulcadir J, Botsikas D, Bolmont M, Bilancioni A, Djema DA, Demicheli FB, Yaron M, Petignat P. Sexual anatomy and function in women with and without genital mutilation - a cross-sectional study. Journal of Sexual Medicine Feb 2016;13(2):226-237 | Assessment of sex/gender was done by asking participants to "check a box" to identify their sex/gender |
|  | Abolhasani M, Maghbouli N, Karbalai Saleh S, Aghsaeifar Z, Sazgara F, Tahmasebi M, et al. Which anthropometric and metabolic index is superior in hypertension prediction among overweight/obese adults? IBPC. 2021 Nov; 14:153–61. | Assessment of sex/gender was done by asking participants to "check a box" to identify their sex/gender |
|  | Abraham AG, Sun J, Sharma A, Yin MT, Brown JK, Demehri S, et al. The combined effects of age and HIV on the anatomic distribution of cortical and cancellous bone in the femoral neck among men and women. AIDS. 2021 Dec 1;35(15):2513–22. | Assessment of sex/gender was done by asking participants to "check a box" to identify their sex/gender |
|  | Achiron R, Pinhas‐Hamiel O, Zalel Y, Rotstein Z, Lipitz S. Development of fetal male gender: prenatal sonographic measurement of the scrotum and evaluation of testicular descent. Ultrasound in Obstet & Gyne. 1998 Apr;11(4):242–5. | Population of study is fetal males |
|  | Adamiak-Godlewska A, Skorupska K, Rechberger T, Romanek-Piva K, Miotła P. Urogynecological and Sexual Functions after Vecchietti Reconstructive Surgery. BioMed Research International. 2019 Feb 25;2019:1–4. | The measurement tool does not link to clinical outcomes |
|  | Addo B, Berchie R. Attitude towards gender norms in Ghana: understanding the dynamics among men and women in intimate relationships. J Pop Research. 2021 Jun;38(2):197–220. | The measurement tool does not link to clinical outcomes |
|  | Adeleye AJ, Reid G, Kao CN, Mok-Lin E, Smith JF. Semen Parameters Among Transgender Women With a History of Hormonal Treatment. Urology. 2019 Feb;124:136–41. | The sex and gender tools are the outcome measures. |
|  | Akasaki M, Ploubidis GB, Dodgeon B, Bonell CP. The clustering of risk behaviours in adolescence and health consequences in middle age. Journal of Adolescence. 2019 Dec;77(1):188–97. | Assessment of sex/gender was done by asking participants to "check a box" to identify their sex/gender |
|  | Akebi T, Saeki S, Hieda H, Goto H. Factors affecting the variability of the torque curves at isokinetic trunk strength testing. Archives of Physical Medicine and Rehabilitation. 1998 Jan;79(1):33–5. | Assessment of sex/gender was done by asking participants to "check a box" to identify their sex/gender |
|  | Allamaneni SSR, Bandaranayake I, Agarwal A. Use of semen quality scores to predict pregnancy rates in couples undergoing intrauterine insemination with donor sperm. Fertility and Sterility. 2004 Sep;82(3):606–11. | Sex/gender measure used by fewer than 2 independent research teams |
|  | Almquist PO, Ekdahl C, Isberg P, Fridén T. Knee rotation in healthy individuals related to age and gender. Journal Orthopaedic Research. 2013 Jan;31(1):23–8. | Assessment of sex/gender was done by asking participants to "check a box" to identify their sex/gender |
|  | Alvisi S, Baldassarre M, Lambertini M, Martelli V, Berra M, Moscatiello S, et al. Sexuality and Psychopathological Aspects in Premenopausal Women with Metabolic Syndrome. The Journal of Sexual Medicine. 2014 Aug 1;11(8):2020–8. | The sex and gender tools are the outcome measures. |
|  | Archer J. Derivation and assessment of a hypermasculine values questionnaire. British J Social Psychol. 2010 Sep;49(3):525–51. | The measurement tool does not link to clinical outcomes |
|  | Assari S, Moazen-Zadeh E, Caldwell CH, Zimmerman MA. Racial Discrimination during Adolescence Predicts Mental Health Deterioration in Adulthood: Gender Differences among Blacks. Front Public Health. 2017 May 29;5:104. | Assessment of sex/gender was done by asking participants to "check a box" to identify their sex/gender |
|  | Azad AD, Charles AG, Ding Q, Trickey AW, Wren SM. The gender gap and healthcare: associations between gender roles and factors affecting healthcare access in Central Malawi, June–August 2017. Arch Public Health. 2020 Dec;78(1):119. | The sex and gender tools are the outcome measures. |
|  | Azizi Z, Gisinger T, Bender U, Deischinger C, Raparelli V, Norris CM, et al. Sex, Gender, and Cardiovascular Health in Canadian and Austrian Populations. Canadian Journal of Cardiology. 2021 Aug;37(8):1240–7. | Sex/gender measure used by fewer than 2 independent research teams |
|  | Bahadori F, Jahanian Sadatmahalleh S, Montazeri A, Nasiri M. Sexuality and psychological well-being in different polycystic ovary syndrome phenotypes compared with healthy controls: a cross-sectional study. BMC Women’s Health. 2022 Sep 25;22(1):390. | The measurement tool does not link to clinical outcomes |
|  | Ballering AV, Bonvanie IJ, Olde Hartman TC, Monden R, Rosmalen JGM. Gender and sex independently associate with common somatic symptoms and lifetime prevalence of chronic disease. Social Science & Medicine. 2020 May;253:112968. | Sex/gender measure used by fewer than 2 independent research teams |
|  | Batalha L, Reynolds KJ, Newbigin CA. All else being equal: Are men always higher in social dominance orientation than women? Euro J Social Psych. 2011 Oct;41(6):796–806. | The measurement tool does not link to clinical outcomes |
|  | Bekker MHJ, van Assen MALM. A short form of the Autonomy Scale - properties of the Autonomy-Connectedness Scale (ACS-30). Journal of Personality Assessment 2006;86(1):51-60 | The measurement tool does not link to clinical outcomes |
|  | Beltz AM. Gendered Mechanisms Underlie the Relation Between Pubertal Timing and Adult Depressive Symptoms. Journal of Adolescent Health. 2018 Jun;62(6):722–8. | Assessment of sex/gender was done by asking participants to "check a box" to identify their sex/gender |
|  | Bender U, Pilote L, Raparelli V. Do sex hormones mediate the effect of gender on acute coronary syndrome outcomes? Canadian Journal of Cardiology 2021;37(2):e8 | Wrong study design – abstract |
|  | Berkery E, Ryan NF. Think manager – Think *male* or *female* : exploring the content of gendered stereotypes of the managerial role among undergraduate business students in Ireland over a 10-year period. GM. 2024 Mar 12;39(3):328–44. | The measurement tool does not link to clinical outcomes |
|  | Bosson JK, Jurek P, Vandello JA, Kosakowska-Berezecka N, Olech M, Besta T, et al. Psychometric Properties and Correlates of Precarious Manhood Beliefs in 62 Nations. Journal of Cross-Cultural Psychology. 2021 Apr;52(3):231–58. | The measurement tool does not link to clinical outcomes |
|  | Bosinski HA, Schroder I, Peter M, Arndt R, Wille R, Sippell WG. Anthropometrical measurements and androgen levels in males, females, and hormonally untreated female-to-male transsexuals. Archives of Sexual Behavior 1997;26(2):143-157 | The measurement tool does not link to clinical outcomes |
|  | Bowman J. Whose unemployment hurts more? Joblessness and subjective well-being in U.S. married couples. Social Science Research. 2023 Mar;111:102795. | Assessment of sex/gender was done by asking participants to "check a box" to identify their sex/gender |
|  | Bowman SJ, Hakeem A, Demant D, McAloon J, Wootton BM. Assessing Gender Dysphoria: Development and Validation of the Gender Preoccupation and Stability Questionnaire – 2^nd^ Edition (GPSQ-2). Journal of Homosexuality. 2024 Feb 23;71(3):666–90. | The measurement tool does not link to clinical outcomes |
|  | Bravo CS, Ayala NPC, Meléndez JC, Almaraz CH. Perfiles de los indicadores relacionados con las disfunciones sexuales masculinas: trastorno de la erección, trastorno del orgasmo y eyaculación precoz. 2010;33(3). | Full text is in Spanish |
|  | Bučková B, Brunovský M, Bareš M, Hlinka J. Predicting Sex From EEG: Validity and Generalizability of Deep-Learning-Based Interpretable Classifier. Front Neurosci. 2020 Oct 27;14:589303. | The measurement tool does not link to clinical outcomes |
|  | Caceres BA, Jackman KB, Belloir J, Dworkin J, Dolezal C, Duncan DT, et al. Examining the associations of gender minority stressors with sleep health in gender minority individuals. Sleep Health. 2022 Apr;8(2):153–60. | Assessment of sex/gender was done by asking participants to "check a box" to identify their sex/gender |
|  | Campos-Castillo C, Shuster SM, Groh SM, Anthony DL. Warning: Hegemonic Masculinity May Not Matter as Much as You Think for Confidant Patterns among Older Men. Sex Roles. 2020 Nov;83(9–10):609–21. | The measurement tool does not link to clinical outcomes |
|  | Casey EA, Masters NT, Beadnell B, Wells EA, Morrison DM, Hoppe MJ. A Latent Class Analysis of Heterosexual Young Men’s Masculinities. Arch Sex Behav. 2016 Jul;45(5):1039–50. | The measurement tool does not link to clinical outcomes |
|  | Castaneda J, Poma N, Mougenot B, Herrera-Añazco P. Association between the Expression of Sexual Orientation and/or Gender Identity and Mental Health Perceptions in the Peruvian LGBTI Population. IJERPH. 2023 Apr 27;20(9):5655. | Assessment of sex/gender was done by asking participants to "check a box" to identify their sex/gender |
|  | Chambers SK, Hyde MK, Oliffe JL, Zajdlewicz L, Lowe A, Wootten AC, Dunn J. Measuring masculinity in the context of chronic disease. Psychology of Men & Masculinities Jul 2016;17(3):228-242 | Sex/gender measure used by fewer than 2 independent research teams |
|  | Chang Q, Yip PSF, Chen YY. Gender inequality and suicide gender ratios in the world. Journal of Affective Disorders. 2019 Jan;243:297–304. | Assessment of sex/gender was done by asking participants to "check a box" to identify their sex/gender |
|  | Chen DR, Chang LY, Yang ML. Gender-specific responses to social determinants associated with self-perceived health in Taiwan: A multilevel approach. Social Science & Medicine. 2008 Nov;67(10):1630–40. | Assessment of sex/gender was done by asking participants to "check a box" to identify their sex/gender |
|  | Choi N. Further examination of the self-efficacy scale. Psychological Reports 2003;92(2):473-480 | The measurement tool does not link to clinical outcomes |
|  | Cho J, Kogan SM. Development and Validation of the Masculine Attributes Questionnaire. Am J Mens Health. 2017 Jul;11(4):941–51. | The measurement tool does not link to clinical outcomes |
|  | Cislaghi B, Bhatia A, Hallgren EST, Horanieh N, Weber AM, Darmstadt GL. Gender Norms and Gender Equality in Full-Time Employment and Health: A 97-Country Analysis of the World Values Survey. Front Psychol. 2022 May 31;13:689815. | The measurement tool does not link to clinical outcomes |
|  | Claridge G, Clark K, Davis C. Nightmares, dreams, and schizotypy. British J Clinic Psychol. 1997 Sep;36(3):377–86. | The measurement tool does not link to clinical outcomes |
|  | Cohen S, Julea C, Goldstein I. 5 Alpha-reductase enzyme deficiency in women - A new syndrome of "female androgen insufficiency". Journal of Sexual Medicine 2014;11(SUPPL. 4):221-222 | Wrong study design - supplement |
|  | Coleman D, Paggi M. Validity and Reliability of a Proxy-Rated Measure of Traditional Masculinity: A Tool For Case-Control and Psychological Autopsy Studies. 2017;8:18. | Sex/gender measure used by fewer than 2 independent research teams |
|  | Colineaux H, Neufcourt L, Delpierre C, Kelly-Irving M, Lepage B. Explaining biological differences between men and women by gendered mechanisms. Emerg Themes Epidemiol. 2023 Mar 23;20(1):2. | The measurement tool does not link to clinical outcomes |
|  | Dacakis G, Oates JM, Douglas JM. Further Evidence of the Construct Validity of the Transsexual Voice Questionnaire (TVQ MtF ) Using Principal Components Analysis. Journal of Voice. 2017 Mar;31(2):142–8. | The measurement tool does not link to clinical outcomes |
|  | Dallal RM, Hatalski A, Trang A, Chernoff A. Longitudinal analysis of cardiovascular parameters after gastric bypass surgery. Surgery for Obesity and Related Diseases. 2012 Nov;8(6):703–9. | Assessment of sex/gender was done by asking participants to "check a box" to identify their sex/gender |
|  | De Breij S, Huisman M, Boot CRL, Deeg DJH. Sex and gender differences in depressive symptoms in older workers: the role of working conditions. BMC Public Health. 2022 Dec;22(1):1023. | Sex/gender measure used by fewer than 2 independent research teams |
|  | DeFroda S, Huddleston H, Williams B, Singh H, Vadhera A, Garrigues G, Nicholson G, Yanke A, Verma N, Cohn M. Do sex-based differences in native glenoid anatomy predispose to shoulder instability? A radiographic analysis. Orthopaedic Journal of Sports Medicine 2022;10():1-5 | Wrong study design – poster |
|  | Diaz FD, Shope MM, Culbert KM. An examination of pubertal timing effects on drive for muscularity in men. Psychology of Men & Masculinities. 2020 Oct;21(4):655–64. | Sex/gender measure used by fewer than 2 independent research teams |
|  | Doyle DM, Begeny CT, Barreto M, Morton TA. Identity-Related Factors Protect Well-Being Against Stigma for Transgender and Gender Non-Conforming People. Arch Sex Behav. 2021 Oct;50(7):3191–200. | Assessment of sex/gender was done by asking participants to "check a box" to identify their sex/gender |
|  | Ehmke T, Aghazadeh M, Bono OJ, Robbins C, Bono JV. Anthropometric measures of the posterior condyles - gender differences and correlation to implant sizing. Journal of Knee Surgery 2021;34(7):679-684 | Assessment of sex/gender was done by asking participants to "check a box" to identify their sex/gender |
|  | Emmanuel AV, Mason HJ, Kamm MA. Relationship between psychological state and level of activity of extrinsic gut innervation in patients with a functional gut disorder. Gut. 2001 Aug 1;49(2):209–13. | Assessment of sex/gender was done by asking participants to "check a box" to identify their sex/gender |
|  | Endut N, Bagheri R, Azwan Azmawati A, Hashimah Mohd Hashim I, Hafizah Selamat N, Mohajer L. The influence of men’s masculine gender-role attitude and behaviour on sexual relationships and reproductive health in Malaysia: A cross-sectional study. IJRM. 2021 Aug 16;19(7):663–70. | The measurement tool does not link to clinical outcomes |
|  | Engler D, Schnabel RB, Neumann FA, Zyriax BC, Makarova N. Sex-Specific Dietary Patterns and Social Behaviour in Low-Risk Individuals. Nutrients. 2023 Apr 11;15(8):1832. | Assessment of sex/gender was done by asking participants to "check a box" to identify their sex/gender |
|  | Esiaka D, Nwakasi C, Brodie K, Philip A, Ogba K. Dying To Be Men: Masculinity and Early Cancer Detection Among Nigerian Men. Community Health Equity Research & Policy. 2022 Jul;42(4):351–9. | The measurement tool does not link to clinical outcomes |
|  | Fisher AD, Bandini E, Casale H, Ferruccio N, Meriggiola MC, Gualerzi A, et al. Sociodemographic and Clinical Features of Gender Identity Disorder: An Italian Multicentric Evaluation. The Journal of Sexual Medicine. 2013 Feb 1;10(2):408–19. | The measurement tool does not link to clinical outcomes |
|  | Foreman M, Hare L, York K, Balakrishnan K, Sánchez FJ, Harte F, et al. Genetic Link Between Gender Dysphoria and Sex Hormone Signaling. The Journal of Clinical Endocrinology & Metabolism. 2019 Feb 1;104(2):390–6. | The measurement tool does not link to clinical outcomes |
|  | Ganguli M, Sun Z. [P4–553]: SOCIAL NORMS AND AGING: EFFECTS OF GENDER AND COGNITION. Alzheimer’s & Dementia [Internet]. 2017 Jul;13(7S_Part_32). Available from: https://alz-journals.onlinelibrary.wiley.com/doi/10.1016/j.jalz.2017.07.716 | Wrong study design – poster abstract |
|  | Gisinger T, Azizi Z, Alipour P, Harreiter J, Raparelli V, Kublickiene K, et al. Sex and gender aspects in diabetes mellitus: Focus on access to health care and cardiovascular outcomes. Front Public Health. 2023 Feb 2;11:1090541. | Sex/gender measure used by fewer than 2 independent research teams |
|  | Glowinska A, Duleba AJ, Zielona-Jenek M, Siakowska M, Pawelczyk L, Banaszewska B. Disparate Relationship of Sexual Satisfaction, Self-Esteem, Anxiety, and Depression with Endocrine Profiles of Women With or Without PCOS. Reprod Sci. 2020 Jan;27(1):432–42. | The sex and gender tools are the outcome measures |
|  | Gómez-Marcos MA, Patino-Alonso MC, Recio-Rodríguez JI, Antón-Alvarez J, Cabrejas-Sánchez A, Fernandez-Alonso C, et al. Confirmatory factor analysis to assess the measure of adiposity that best fits the diagnosis of metabolic syndrome and relationship to physical activity in adults. Eur J Nutr. 2013 Aug;52(5):1451–9. | Assessment of sex/gender was done by asking participants to "check a box" to identify their sex/gender |
|  | Green MA, Davids CM, Skaggs AK, Riopel CM, Hallengren JJ. Femininity and Eating Disorders. Eating Disorders. 2008 Jul 4;16(4):283–93. | Sex/gender measure used by fewer than 2 independent research teams |
|  | Gul B, Lansky A, Budoff MJ, Sharp D, Maniet B, Herman L, et al. The Clinical Utility of a Precision Medicine Blood Test Incorporating Age, Sex, and Gene Expression for Evaluating Women with Stable Symptoms Suggestive of Obstructive Coronary Artery Disease: Analysis from the PRESET Registry. Journal of Women’s Health. 2019 May;28(5):728–35. | Assessment of sex/gender was done by asking participants to "check a box" to identify their sex/gender |
|  | Gustavsson JP, Eriksson A, Hilding A, Gunnarsson M, Östensson C. Measurement invariance of personality traits from a five‐factor model perspective: multi‐group confirmatory factor analyses of the HP5 inventory. Scandinavian J Psychology. 2008 Oct;49(5):459–67. | Assessment of sex/gender was done by asking participants to "check a box" to identify their sex/gender |
|  | Hakeem A, Crncec R, Asghari-Fard M, Harte F, Eapen V. Development and validation of a measure for assessing gender dysphoria in adults - The Gender Preoccupation and Stability Questionnaire. International Journal of Transgenderism 2016;17(3-4):131-140 | Sex/gender measure used by fewer than 2 independent research teams |
|  | Hall JA. An Exploratory Study of Communication, Gender-Role Conflict, and Social Support of Parents of Children Treated at Children’s Hospital. Journal of Psychosocial Oncology. 2010 Aug 24;28(5):511–25. | The measurement tool does not link to clinical outcomes |
|  | Hardy TLD, Rieger JM, Wells K, Boliek CA. Associations Between Voice and Gestural Characteristics of Transgender Women and Self-Rated Femininity, Satisfaction, and Quality of Life. Am J Speech Lang Pathol. 2021 Mar 26;30(2):663–72. | The measurement tool does not link to clinical outcomes |
|  | Hee Jeong K. The effect of adult attachment types, gender role attitude on the heterosexual relationship satisfaction in university students. Medico-Legal Update 2020;20(1):1552-1557 | The measurement tool does not link to clinical outcomes |
|  | Hoyt MA, Stanton AL, Irwin MR, Thomas KS. Cancer-related masculine threat, emotional approach coping, and physical functioning following treatment for prostate cancer. Health Psychology. 2013;32(1):66–74. | The measurement tool does not link to clinical outcomes |
|  | Huang CY, Liou CF, Lee SH, Tsai LY. The Relationship Between Gender Role Orientation and Sexual Health Care in Taiwanese Nurses: A Structural Equation Model. Sexual Medicine. 2020 Sep 1;8(3):565–73. | The measurement tool does not link to clinical outcomes |
|  | Huang CL, Chen SY, Hsu HC, Wang RH. Scale for Measuring Role Strain in Women With Diabetes: Development and Psychometric Testing of the Chinese Version. J Cardiovasc Nurs. 2020 Sep;35(5):483–90. | The measurement tool does not link to clinical outcomes |
|  | Hunt K. Contextualizing smoking: masculinity, femininity and class differences in smoking in men and women from three generations in the west of Scotland. Health Education Research. 2004 Jun 1;19(3):239–49. | The measurement tool does not link to clinical outcomes |
|  | Hunt K. A generation apart? Gender-related experiences and health in women in early and late mid-life. Social Science & Medicine. 2002 Mar;54(5):663–76. | The measurement tool does not link to clinical outcomes |
|  | Jacobson LE, Daire AP, Abel EM, Lambie G. Gender Expression Differences in Same-Sex Intimate Partner Violence Victimization, Perpetration, and Attitudes among LGBTQ College Students. Journal of LGBT Issues in Counseling. 2015 Jul 3;9(3):199–216. | The measurement tool does not link to clinical outcomes |
|  | Jones TV. Predictors of perceptions of mental illness and averseness to help: a survey of elite football players. Journal of Mental Health. 2016 Sep 2;25(5):422–7. | The measurement tool does not link to clinical outcomes |
|  | Kellett P, O’Lynn CE, Herakova LL, O’Connor T. Gender Role Conflict and Male Nursing Students’ Academic and Program Success. J Nurs Educ. 2023 Jan;62(1):42–6. | Wrong study design – research brief |
|  | Kendler KS, Gardner CO, Prescott CA. A population-based twin study of self-esteem and gender. Psychol Med. 1998 Nov;28(6):1403–9. | Assessment of sex/gender was done by asking participants to "check a box" to identify their sex/gender |
|  | Kerr P, Barbosa Da Torre M, Giguere CE, Lupien S, Juster RP. Occupational gender-roles in relation to allostatic load, workplace stress and mental health of psychiatric hospital workers. Psychoneuroendocrinology. 2020;119(Supplement): 2020. | Wrong study design - supplement |
|  | Khairallah, T.S., Treloar, H.R., McCarthy, D.M. The influence of gender role adherence on alcohol use and problems. Samuel B. Guze Symposium on Alcoholism. 2010:Poster 19. | Wrong study design - poster |
|  | Kim C, Teo C, Nielsen A, Chum A. Macro-level gender equality and women’s depressive symptoms in South Korea: a longitudinal study. Soc Psychiatry Psychiatr Epidemiol. 2023 Mar;58(3):383–93. | The measure does not include gender roles, behaviors, expressions |
|  | Koc Y, Vignoles VL. Global identification predicts gay–male identity integration and well‐being among Turkish gay men. British J Social Psychol. 2016 Dec;55(4):643–61. | The measurement tool does not link to clinical outcomes |
|  | Komon W, Kijmanawat A, Chattrakulchai K, et al. Validation of the Thai version of the Female Genital Self-Image Scale (FGSIS). BMC Women’s Health. 2022;22:254. | The measurement tool does not link to clinical outcomes |
|  | Krupp K, Brunner F, Fliegner M, Rall K, Brucker S, Briken P, Richter-Appelt H, A questionnaire for the assessment of women's perception of their own femininity - a study on women with Mayer-Rokitansky-Kuster-Hauser-syndrome and women with polycystic ovary syndrome. Psychotherapie, Psychosomatik, Medizinische Psychologie 2013;63(8):334-340 | Full text is in German |
|  | Kubicka, L. & Csemy, L. Women’s gender role orientation predicts their drinking patterns: A follow-up study of Czech women. Addiction. 2008;103(6):929-937. | The measurement tool does not link to clinical outcomes |
|  | Kuntsche S, Kuntsche E. Being old fashioned in a modern world: Gender role attitudes moderate the relation between role conflicts and alcohol use of parents. Drug and Alcohol Dependence. 2019 Feb;195:90–3. | The measurement tool does not link to clinical outcomes |
|  | Lake JE, Feng H, Miao H, Debroy P, Dobs A, Haberlen S, Kingsley L, et al. Burden of coronary disease in transgender women with and without HIV. Topics in Antiviral Medicine 2023;31(2):254 | Wrong study design - abstract |
|  | Langelier DM, Cormie P, Bridel W, Grant C, Albinati N, Shank J, et al. Perceptions of masculinity and body image in men with prostate cancer: the role of exercise. Support Care Cancer. 2018 Oct;26(10):3379–88. | The sex and gender tools are the outcome measures. |
|  | Lansky A, Elashoff MR, Ng V, McPherson J, Lazar D, Kraus WE, et al. A gender-specific blood-based gene expression score for assessing obstructive coronary artery disease in nondiabetic patients: Results of the Personalized Risk Evaluation and Diagnosis in the Coronary Tree (PREDICT) Trial. American Heart Journal. 2012 Sep;164(3):320–6. | Sex/gender measure used by fewer than 2 independent research teams |
|  | Lazzeri M, Lughezzani G, Haese A, McNicholas T, De La Taille A, Buffi NM, et al. Clinical performance of prostate health index in men with tPSA>10ng/ml: Results from a multicentric European study. Urologic Oncology: Seminars and Original Investigations. 2016 Sep;34(9):415.e13-415.e19. | Sex/gender measure used by fewer than 2 independent research teams |
|  | Lee JP, You D, Jeong IG, Hong BS, Hong JH, Kim CS, Ahn G, Song C. Associations of sarcopenia on survival of patients with organ confined renal cell carcinoma after radical nephrectomy. International Journal of Urology 2020;27(SUPPL 1):107-108 | Wrong study design - supplement |
|  | Leineweber C, Falkenberg H, Albrecht SC. Parent’s Relative Perceived Work Flexibility Compared to Their Partner Is Associated With Emotional Exhaustion. Front Psychol. 2018 May 3;9:640. | Assessment of sex/gender was done by asking participants to "check a box" to identify their sex/gender |
|  | Levinsson A, De Denus S, Sandoval J, Lemieux Perreault LP, Rouleau J, Tardif JC, et al. Construction of a femininity score in the UK Biobank and its association with angina diagnosis prior to myocardial infarction. Sci Rep. 2022 Feb 2;12(1):1780. | Sex/gender measure used by fewer than 2 independent research teams |
|  | Lewis JA, Neville HA. Construction and initial validation of the Gendered Racial Microaggressions Scale for Black women. Journal of Counseling Psychology. 2015 Apr;62(2):289–302. | Assessment of sex/gender was done by asking participants to "check a box" to identify their sex/gender |
|  | Li Y, Wang W, Lin P, Lu H, Zhang Y, Li Q, Zhang Z. Study on the psychologic status and personality traits of patients with obstructive sleep apnea hypopnea syndrome. Zhonghua er bi yan hou tou jing wai ke za zhi 2015;50(7):587-593 | Full text is in Chinese |
|  | Lorello GR, Peel JK, Cygler J, Kirkham K, Flexman AM. Standing out or fitting in - a latent-pattern content analysis of discrimination of women and 2SLGBTQ+ anesthesiologists. Canadian Journal of Anesthesia. 2022;69(Supplement 2):S200-S201 | Wrong study design – supplement |
|  | Lu Y-M, Chen C-H, & Lue Y-J. A cross-sectional study of disability and quality of life in patients with low back pain: Focus on sex and gender. Journal of Back and Musculoskeletal Rehabilitation. 2022;35:177-184. | The measurement tool does not link to clinical outcomes |
|  | Lu ZH, Yu JK, Chen LX, Gong X, Wang YJ, Leung KKM. Computed Tomographic Measurement of Gender Differences in Bowing of the Sagittal Femoral Shaft in Persons Older Than 50 Years. The Journal of Arthroplasty. 2012 Jun;27(6):1216–20. | Assessment of sex/gender was done by asking participants to "check a box" to identify their sex/gender |
|  | Luckhoff HK, Asmal L, Scheffler F, Du Plessis S, Buckle C, Chiliza B, et al. Gender role endorsement in first-episode schizophrenia spectrum disorders. Psychiatry Research. 2021 May;299:113867. | The measurement tool does not link to clinical outcomes |
|  | Manlove HA, Guillermo C, Gray PB. Do women with polycystic ovary syndrome (PCOS) report differences in sex-typed behavior as children and adolescents?: Results of a pilot study. Annals of Human Biology. 2008 Jan;35(6):584–95. | The measurement tool does not link to clinical outcomes |
|  | Mansdotter A, Lundin A, Falkstedt D, Hemmingsson T. The association between masculinity rank and mortality patterns: a prospective study based on the Swedish 1969 conscript cohort. Journal of Epidemiology & Community Health. 2009 May 1;63(5):408–13. | Sex/gender measure used by fewer than 2 independent research teams |
|  | McHale SM, Huston TL. Men and Women as Parents: Sex Role Orientations, Employment, and Parental Roles with Infants. Child Development. 1984 Aug;55(4):1349. | The measurement tool does not link to clinical outcomes |
|  | McLaughlin H, Uggen C, Blackstone A. Sexual Harassment, Workplace Authority, and the Paradox of Power. Am Sociol Rev. 2012 Aug;77(4):625–47. | The measurement tool does not link to clinical outcomes |
|  | McLaughlin K, Muldoon OT, Moutray M. Gender, gender roles and completion of nursing education: A longitudinal study. Nurse Education Today. 2010 May;30(4):303–7. | The measurement tool does not link to clinical outcomes |
|  | Melo AS, Vieira Macedo CS, Maltoni Romano LG, Ferriani RG, de Albuquerque Salles Navarro PA. Women with polycystric ovary syndrome have a higher frequency of metabolic syndrome regardless of body mass index. Revista Brasileira De Ginecologia E Obstetricia Jan 2012;34(1):4-10 | Full text is in Portuguese |
|  | Mendoza-Catalán G, D’Alonzo K, Domínguez-Chávez CJ, Villa-Rueda AA, González-Ramírez J. Relationship of *Marianismo* Beliefs With the Self-Care Behaviors of Mexican Women With Type 2 Diabetes Mellitus. J Transcult Nurs. 2023 Mar;34(2):151–6. | Sex/gender measure used by fewer than 2 independent research teams |
|  | Menon R, Di Dario M, Cordiglieri C, Musio S, La Mantia L, Milanese C, et al. Gender-based blood transcriptomes and interactomes in multiple sclerosis: Involvement of SP1 dependent gene transcription. Journal of Autoimmunity. 2012 May;38(2–3):J144–55. | The sex and gender tools are the outcome measures. |
|  | Menon V, Breyer B, Copp HL, Baskin L, Disandro M, Schlomer BJ. Do adult men with untreated ventral penile curvature have adverse outcomes? Journal of Pediatric Urology. 2016 Feb;12(1):31.e1-31.e7. | The sex and gender tools are the outcome measures. |
|  | Milner A, Kavanagh A, King T, Currier D. The Influence of Masculine Norms and Occupational Factors on Mental Health: Evidence From the Baseline of the Australian Longitudinal Study on Male Health. Am J Mens Health. 2018 Jul;12(4):696–705. | The measurement tool does not link to clinical outcomes |
|  | Milner A, Shields M, King T. The Influence of Masculine Norms and Mental Health on Health Literacy Among Men: Evidence From the Ten to Men Study. Am J Mens Health. 2019 Oct;13(5):1557988319873532. | The measurement tool does not link to clinical outcomes |
|  | Moreira IFDA, Bianchini MP, Moreira GRC, Almeida AM, Rezende BA. Sexual function and metabolic/hormonal changes in women using long-term hormonal and non-hormonal contraceptives: a pilot study. BMC Women’s Health. 2020 Dec;20(1):240. | The sex and gender tools are the outcome measures. |
|  | Mueller SC, Wierckx K, Jackson K, T’Sjoen G. Circulating androgens correlate with resting-state MRI in transgender men. Psychoneuroendocrinology. 2016 Nov;73:91–8. | The measurement tool does not link to clinical outcomes |
|  | Nicdomhnaill OM. Understanding gender inequality in employment - The impact of sex and gender identity on organizational citizenship behaviors and its proposed impact on compensation, promotion and burnout. Dissertation Abstracts International Section B The Sciences & Engineering 2007;67(10-B):6104 | Wrong study design – abstract |
|  | Niemeier J, Hurst B, Foureau D. Effects of circulating reproductive hormones and traumatic brain injury (TBI) onset on short-term TBI outcomes for women ages 18-35. Archives of Physical Medicine & Rehabilitation 2016;97(10):e69 | Wrong study design – abstract |
|  | Norris CM, Johnson NL, Hardwicke-Brown E, McEwan M, Pelletier R, Pilote L. The Contribution of Gender to Apparent Sex Differences in Health Status Among Patients with Coronary Artery Disease. Journal of Women’s Health. 2017 Jan;26(1):50–7. | Sex/gender measure used by fewer than 2 independent research teams |
|  | Nowotny KM, Peterson RL, Boardman JD. Gendered contexts: Variation in suicidal ideation by female and male youth across U.S. states. Journal of Health and Social Behavior. 2015;56(1):114-130 | Sex/gender measure used by fewer than 2 independent research teams |
|  | O’Brien KS, Forrest W, Greenlees I, Rhind D, Jowett S, Pinsky I, et al. Alcohol consumption, masculinity, and alcohol-related violence and anti-social behaviour in sportspeople. Journal of Science and Medicine in Sport. 2018 Apr;21(4):335–41. | The measurement tool does not link to clinical outcomes |
|  | Occhipinti S, Laurie K, Hyde MK, Martin S, Oliffe J, Wittert G, et al. Measuring Masculinity in Men With Chronic Disease. Am J Mens Health. 2019 Jul;13(4):1557988319859706. | Sex/gender measure used by fewer than 2 independent research teams |
|  | Onder M, Durak Batigun A. Premature and normal menopause: an evaluation in terms of stress, marital adjustment and sex roles. Dusunen Adam. 2016 Jun 28;129–38. | The measurement tool does not link to clinical outcomes |
|  | Pappadis MR, Sander AM, Leung P, Parrish DE, Epstein MW. Impact of gender role conflict on adjustment in women with traumatic brain injury. Archives of Physical Medicine & Rehabilitation 2014;95(10):e68-e69 | Wrong study design - abstract |
|  | Parent MC, Moradi B. An abbreviated tool for assessing feminine norm conformity - psychometric properties of the Conformity to Feminine Norms Inventory-45. Psychological Assessment 2011;23(4):958-969 | The measurement tool does not link to clinical outcomes |
|  | Park S, Kang Y, Surkan PJ. Types of social capital in relation to self-rated health - Gender differences in a nationally representative cross-sectional study of South Korean adults. Global Public Health An International Journal for Research, Policy & Practice 2022;17(7):1365-1378 | Assessment of sex/gender was done by asking participants to "check a box" to identify their sex/gender |
|  | Peel JK, Flexman AM, Cygler J, Kirkham KR, Lorello GR. Standing out or fitting in: A latent projective content analysis of discrimination of women and 2SLGBTQ+ anesthesiologists and providers. Journal of Clinical Anesthesia. 2022 Sep;80:110884. | The measurement tool does not link to clinical outcomes |
|  | Pelletier R, Ditto B, Pilote L. A Composite Measure of Gender and Its Association With Risk Factors in Patients With Premature Acute Coronary Syndrome. Psychosomatic Medicine. 2015 Jun;77(5):517–26. | Sex/gender measure used by fewer than 2 independent research teams |
|  | Pelletier R, Khan NA, Cox J, Daskalopoulou SS, Eisenberg MJ, Bacon SL, et al. Sex Versus Gender-Related Characteristics. Journal of the American College of Cardiology. 2016 Jan;67(2):127–35. | Sex/gender measure used by fewer than 2 independent research teams |
|  | Petit F, Hubert-Buron A, Mollet-Boudjemline A, Sechepine A, Milcent K, Guyonnet C, et al. Mise au point et validation d’un outil d’évaluation de la santé sexuelle sous forme d’auto-questionnaires pour une application aux maladies métaboliques. Progrès en Urologie. 2013 Mar;23(3):210–8. | Full text is in French |
|  | Pinho-Gomes AC, Vassallo A, Carcel C, Peters S, Woodward M. Gender equality and the gender gap in life expectancy in the European Union. BMJ Glob Health. 2022 Feb;7(2):e008278. | The measurement tool does not link to clinical outcomes |
|  | Pokrywka G, Ladapo JA, Wright R, McLaughlin P, Maniet B, Sharp D, Ross L, Huang L, Monane M, Budoff M. The clinical utility of a blood test incorporating age, sex, and gene expression in the evaluation of women presenting with stable symptoms suggestive of obstructive coronary artery disease in a large primary care registry (PRESET) - Subgroup analysis of the primary efficacy endpoint. Menopause 2015;22(12):1400 | Wrong study design - abstract |
|  | Rakic Z, Starcevic V, Maric J, Kelin K. The outcome of sex reassignment surgery in Belgrade: 32 patients of both sexes. Arch Sex Behav. 1996 Oct;25(5):515–25. | There is not a tool or scale that measures sex or gender |
|  | Ramirez K, Fernández R, Collet S, Kiyar M, Delgado-Zayas E, Gómez-Gil E, et al. Epigenetics Is Implicated in the Basis of Gender Incongruence: An Epigenome-Wide Association Analysis. Front Neurosci. 2021 Aug 19;15:701017. | The measurement tool does not link to clinical outcomes |
|  | Rao K, Apte M, Subbakrishna DK. Coping and Subjective Wellbeing in Women with Multiple Roles. Int J Soc Psychiatry. 2003 Sep;49(3):175–84. | There is not a tool or scale that measures sex or gender |
|  | Reilley JL. The educational participation of the female registered nurse baccalaureate student - motivation, barriers, and persistence to complete the degree. 2003;():114. | Wrong study design – dissertation |
|  | Rhead R, Skovdal M, Takaruza A, Maswera R, Nyamukapa C, Gregson S. The multidimensionality of masculine norms in east Zimbabwe: implications for HIV prevention, testing and treatment. AIDS. 2019 Mar 1;33(3):537–46. | Sex/gender measure used by fewer than 2 independent research teams |
|  | Saelee R, Vaccarino V, Sullivan S, Hammadah M, Shah A, Wilmot K, et al. Longitudinal associations between self-reported experiences of discrimination and depressive symptoms in young women and men post- myocardial infarction. Journal of Psychosomatic Research. 2019 Sep;124:109782. | Assessment of sex/gender was done by asking participants to "check a box" to identify their sex/gender |
|  | Sanchez Bravo C, Corres Ayala NP, Carreno Melendez J, Henales Almaraz C. Profiles of the indicators related with male sexual dysfunctions - erectile dysfunction, male orgasmic disorder and precocious ejaculation. Salud Mental May-Jun 2010;33(3):237-242 | Full text is in Spanish |
|  | Sawyer KS, Oscar‐Berman M, Mosher Ruiz S, Gálvez DA, Makris N, Harris GJ, et al. Associations Between Cerebellar Subregional Morphometry and Alcoholism History in Men and Women. Alcoholism Clin &amp; Exp Res. 2016 Jun;40(6):1262–72. | Assessment of sex/gender was done by asking participants to "check a box" to identify their sex/gender |
|  | Schmalzing M, Nau LF, Gernert M, Froehlich M, Schwaneck EC. Sexual function in German women with systemic sclerosis compared to women with systemic lupus erythematosus and evaluation of a screening test. Clinical and Experimental Rheumatology. 2020; | The sex and gender tools are the outcome measures |
|  | Schopp LH, Good GE, Barker KB, Mazurek MO, Hathaway SL. Masculine role adherence and outcomes among men with traumatic brain injury. Brain Injury. 2006 Jan;20(11):1155–62. | The measurement tool does not link to clinical outcomes |
|  | Shapiro JR, Seddu K, Park HS, Lee JS, Creisher PS, Yin A, et al. The intersection of biological sex and gender in adverse events following seasonal influenza vaccination in older adults [Internet]. 2023 [cited 2025 Jun 21]. Available from: https://www.researchsquare.com/article/rs-2557775/v1 | The measurement tool does not link to clinical outcomes |
|  | Sullivan L, Camic PM, Brown JSL. Masculinity, alexithymia, and fear of intimacy as predictors of UK men’s attitudes towards seeking professional psychological help. British J Health Psychol. 2015 Feb;20(1):194–211. | Sex/gender measure used by fewer than 2 independent research teams |
|  | Sutter B, Fehr M, Hartmann C, Schmid S, Zitzmann M, Stute P. Androgen receptor gene polymorphism and sexual function in midlife women. Arch Gynecol Obstet. 2019 Apr;299(4):1173–83. | Sex/gender measure used by fewer than 2 independent research teams |
|  | Tabler J, Schmitz RM, Nagata JM, Geist C. Self-perceived gender expression, discrimination, and mental health disparities in adulthood. SSM - Mental Health. 2021 Dec;1:100020. | Sex/gender measure used by fewer than 2 independent research teams |
|  | Tharoor H, Mohan G, Gopal S. Sex hormones and psychopathology in drug naïve Schizophrenia. Asian Journal of Psychiatry. 2020 Aug;52:102042. | The measurement tool does not link to clinical outcomes |
|  | Thomas TR, Tener AJ, Pearlman AM, Imborek KL, Yang JS, Strang JF, et al. Polygenic Scores Clarify the Relationship Between Mental Health and Gender Diversity. Biological Psychiatry Global Open Science. 2024 Mar;4(2):100291. | Sex/gender measure used by fewer than 2 independent research teams |
|  | Topa G, Segura A, Pérez S. Gender differences in retirement planning: A longitudinal study among Registered Nurses. J Nurs Manag. 2018 Jul;26(5):587–96. | The tool or scale does not measure sex or gender |
|  | Topîrceanu A, Udrescu L, Udrescu M, Mihaicuta S. Gender Phenotyping of Patients with Obstructive Sleep Apnea Syndrome Using a Network Science Approach. JCM. 2020 Dec 12;9(12):4025. | Assessment of sex/gender was done by asking participants to "check a box" to identify their sex/gender |
|  | Ullman J, Hobby L, Magson NR, Zhong HF. Students’ perceptions of the rules and restrictions of gender at school: A psychometric evaluation of the Gender Climate Scale (GCS). Front Psychol. 2023 Mar 2;14:1095255. | Sex/gender measure used by fewer than 2 independent research teams |
|  | Vader SS, Lewis SM, Verdonk P, Verschuren WMM, Picavet HSJ. Masculine gender affects sex differences in the prevalence of chronic health problems - The Doetinchem Cohort Study. Preventive Medicine Reports. 2023 Jun;33:102202. | Sex/gender measure used by fewer than 2 independent research teams |
|  | Van Well S, Kolk AM, Arrindell WA. Cross-Cultural Validity of the Masculine and Feminine Gender Role Stress Scales. Journal of Personality Assessment. 2005 Jun;84(3):271–8. | The measurement tool does not link to clinical outcomes . |
|  | Vyas S, Jansen HAFM, Viet NNT, Gardner J, Loan TTB, Phan H. Changes in the Correlates for Intimate Partner Violence Against Women in Vietnam: Evidence From the 2010 and 2019 National Prevalence Surveys. Violence Against Women. 2023 Nov;29(14):2699–729. | The measurement tool does not link to clinical outcomes . |
|  | Wacker E, Fischer A, Schorlemmer J. Effects of person-environment fit of gender-role orientation on burnout, engagement and hair steroids as stress biomarkers among women. J Occup Med Toxicol. 2021 Dec;16(1):13. | Sex/gender measure used by fewer than 2 independent research teams |
|  | Walcott MM, Ehiri J, Kempf MC, Funkhouser E, Bakhoya M, Aung M, et al. Gender Norms and Family Planning Practices Among Men in Western Jamaica. Am J Mens Health. 2015 Jul;9(4):307–16. | Sex/gender measure used by fewer than 2 independent research teams |
|  | Wu Y, Wang L, Su Y, Cheng XG. Is vitamin D associated with muscle and bone mineral density in elderly women and men with low-energy hip fractures? Osteoporosis Intervational. 2017;28(Supplement 1):S565 | Wrong study design – supplement |
|  | Yang X, Lau JTF, Wang Z, Lau MCM. Prevalence of binge drinking and relationships between masculine role discrepancy and binge drinking via discrepancy stress among Chinese men. Drug and Alcohol Dependence. 2019 Mar;196:57–61. | The measurement tool does not link to clinical outcomes |
|  | Yu S, Appleton S, Chapman I, Adams R, Wittert G, Visvanathan T, et al. An Anthropometric Prediction Equation for Appendicular Skeletal Muscle Mass in Combination With a Measure of Muscle Function to Screen for Sarcopenia in Primary and Aged Care. Journal of the American Medical Directors Association. 2015 Jan;16(1):25–30. | Assessment of sex/gender was done by asking participants to "check a box" to identify their sex/gender |
|  | Zhang Y, Li T, Reddy A, Nallasamy N. Gender differences in refraction prediction error of five formulas for cataract surgery. BMC Ophthalmol. 2021 Dec;21(1):183. | Assessment of sex/gender was done by asking participants to "check a box" to identify their sex/gender |
