# Supplementary material for: The effects of sex and gender attributes on clinical outcomes: a systematic review
Source: Biol Sex Differ. 2025 Dec 29;16:108. doi: 10.1186/s13293-025-00772-x (PMC12751436; doi:10.1186/s13293-025-00772-x)
Supplement: Supplementary file 5 — Supplementary Material 5. [file 13293_2025_772_MOESM5_ESM.docx]

**Supplement 5**

**Supplement 5a**. QUIPS Six Potential Sources of Bias and assessment criteria

| **QUIPS Six Potential Sources of Bias** | | **Assessment Criteria** | |
| --- | --- | --- | --- |
| 1. | Study Design | 1. | Was the research question or objective in this paper clearly stated? |
|  |  | 10. | Was the exposure(s) assessed more than once over time? |
|  |  | 12. | Were the outcome assessors blinded to the exposure status of participants? |
| 2. | Study Participation | 2. | Was the study population clearly specified and defined?  *Authors reported age and at least one PROGRESS Plus parameter, in addition to the exposure variable: place of residence, race/ethnicity/culture/language, occupation, religion, education, socioeconomic status, social capital, contextual parameters within PLUS such as disability, sexual orientation, indigeneity.* |
|  |  | 3. | Was the participation rate of eligible persons at least 50%? |
|  |  | 4. | Were all the subjects selected or recruited from the same or similar populations (including the same time period)? Were inclusion and exclusion criteria for being in the study prespecified and applied uniformly to all participants? |
| 3. | Study Attrition | 13. | Was loss to follow-up after baseline 20% or less? |
| 4. | Associated Factors | 9. | Were the exposure measures (independent variables) clearly defined, valid, reliable, and implemented consistently across all study participants? |
| 5. | Outcome Measures/ Confounding Account | 11. | Were the outcome measures (dependent variables) clearly defined, valid, reliable, and implemented consistently across all study participants? |
|  |  | 14. | Were key potential confounding variables measured and adjusted statistically for their impact on the relationship between exposure(s) and outcome(s)? *Authors considered (at least) key potential confounding variable age.* |
| 6. | Analysis | 5. | Was a sample size justification, power description, or variance and effect estimates provided? |
|  |  | 6. | For the analyses in this paper, were the exposure(s) of interest measured prior to the outcome(s) being measured? |
|  |  | 7. | Was the timeframe sufficient so that one could reasonably expect to see an association between exposure and outcome if it existed? |
|  |  | 8. | For exposures that can vary in amount or level, did the study examine different levels of the exposure as related to the outcome (e.g., categories of exposure, or exposure measured as continuous variable)? |

**Supplement 5b.** Summary of scoring

| **1. Crude Score** | Each Assessment Criteria was rated as yes (Y), no (N), cannot determine (CD), not reported (NR), or not applicable (NA). The crude score was calculated by dividing the number of 'yes' (Y) responses by the total number of applicable criteria. Criteria rated as not applicable (NA) were excluded from the total. |
| --- | --- |
| **2. Source of Bias Rating** | Each Source of Bias was rated as “+”, “-”, or not applicable (NA):   - **“+”** when most Assessment Criteria were rated as yes (Y) in that Source of Bias category. - **“-”** when one or more Assessment Criteria were rated as no (N) in that Source of Bias category. - **Not applicable (NA)** when all Assessment Criteria in that Source of Bias category were rated as NA. |
| **3. Overall Rating** | Finally, each study quality was summarized qualitatively into three groups:   - **Excellent (“++”)** when all or most of the Sources of Bias were rated as “+”. Studies that had more than one Assessment Criteria rated as not reported (NR) or cannot determine (CD) cannot be as rated “++”. - **Good (“+”)** when more than 50% of the Sources of Bias were rated as “+”. - **Fair (“-”)** when 50% or more of the Sources of Bias were rated as “-”. |

**Supplement 5c**. Quality assessment of included studies using the QUIPS Six Potential Sources of Bias

| **Study** | **Assessment Criteria** | | | | | | | | | | | | | | **Crude Score** | **Source of Bias Rating** | | | | | | | | | | | **Overall Rating** | |  |
| --- | --- | --- | --- | --- | --- | --- | --- | --- | --- | --- | --- | --- | --- | --- | --- | --- | --- | --- | --- | --- | --- | --- | --- | --- | --- | --- | --- | --- | --- |
|  | **1** | **2** | **3** | **4** | **5** | **6** | **7** | **8** | **9** | **10** | **11** | **12** | **13** | **14** |  | **1** | **2** | **3** | | **4** | | | **5** | | **6** | |  |  |  |
| Arcand et al. 2023 | Y | N | NR | Y  (Y/Y) | Y | Y | CD | Y | CD | N | CD | NA | Y | N | 6/13 | - | - | + | | - | | | - | | + | | **Fair**  **-** | |  |
| Boeri et al., 2017 | Y | Y | NR | Y  (Y/Y) | Y | NR | NA | Y | Y | NA | Y | NA | NA | Y | 8/10 | + | + | NA | | + | | | + | | - | | **Good**  **+** | |  |
| Cunningham et al. 2020 | Y | Y | NR | N  (NR/N) | Y | Y | NA | N | Y | NA | Y | NA | NA | N | 6/10 | + | - | NA | | + | | | - | | + | | **Good**  **+** | |  |
| Dumesic et al., 2019 | Y | Y | NR | Y  (Y/Y) | Y | NR | NA | Y | Y | NA | Y | NA | NA | Y | 8/10 | + | + | NA | | + | | | + | | - | | **Good**  **+** | |  |
| Gibson et al., 2015 | Y | Y | NR | Y  (Y/Y) | Y | Y | NA | N | Y | NA | CD | NA | NA | Y | 7/10 | + | + | NA | | + | | | - | | - | | **Good**  **+** | |  |
| Helgeson, 1991 | Y | Y | Y | Y  (Y/Y) | Y | Y | CD | N | Y | N | Y | NA | Y | N | 9/13 | - | + | + | | + | | | - | | - | | **Fair**  **-** | |  |
| Hunt et al., 2006 | Y | Y | NR | Y  (Y/Y) | Y | Y | CD | N | Y | N | N | NA | NR | N | 6/13 | + | + | - | | - | | | - | | - | | **Fair**  **-** | |  |
| Hunt et al., 2007 | Y | Y | Y | Y  (Y/Y) | Y | Y | NA | Y | Y | NA | Y | NA | NA | N | 9/10 | + | + | NA | | + | | | - | | + | | **Good**  **+** | |  |
| Iwamoto et al., 2018 | Y | Y | NR | Y  (Y/Y) | Y | Y | CD | Y | Y | N | Y | NA | Y | N | 9/13 | - | + | + | | + | | | - | | + | | **Good**  **+** | |  |
| Kerr et al., 2021 | Y | Y | N | Y  (Y/Y) | Y | Y | NA | N | Y | NA | Y | NA | NA | N | 7/10 | + | + | NA | | + | | | - | | - | | **Good**  **+** | |  |
| Leinonen et al., 2023 | Y | Y | Y | Y  (Y/Y) | Y | Y | NA | Y | Y | Y | Y | NA | NA | N | 10/11 | + | + | NA | | + | | | - | | + | | **Good**  **+** | |  |
| Moller-Leimkuhler, 2010 | Y | Y | NR | Y  (Y/Y) | Y | Y | NA | Y | Y | NA | Y | NA | NA | N | 8/10 | + | + | NA | | + | | | - | | + | | **Good**  **+** | |  |
| Nguefack et al., 2022 | Y | Y | Y | Y  (Y/Y) | Y | Y | NA | Y | Y | NA | Y | NA | NA | Y | 10/10 | + | + | NA | | + | | | + | | + | | **Excellent**  **++** | |  |
| Po Yee Lo et al., 2019 | Y | Y | N | Y  (Y/Y) | Y | Y | NA | Y | Y | NA | Y | NA | NA | Y | 9/10 | + | + | NA | | + | | | + | | + | | **Excellent**  **++** | |  |
| Short et al., 2023 | Y | Y | NR | Y  (Y/Y) | Y | Y | NA | N | Y | NA | Y | NA | NA | Y | 8/10 | + | + | NA | | + | | | + | | - | | **Good**  **+** | |  |
| Snyder et al., 2016 | Y | N | NR | Y  (NR/Y) | Y | Y | Y | Y | Y | Y | Y | Y | Y | Y | 12/14 | + | - | + | | + | | | + | | + | | **Good**  **+** | |  |
| Vafaei et al., 2016 | Y | Y | Y | Y  (Y/Y) | Y | Y | NA | Y | Y | NA | Y | NA | NA | Y | 10/10 | + | + | NA | | + | | | + | | + | | **Excellent**  **++** | |  |
| Yang et al., 2018 | Y | Y | Y | Y  (Y/Y) | Y | Y | NA | N | CD | NA | Y | NA | NA | Y | 8/10 | + | + | NA | | - | | | + | | - | | **Good**  **+** | |  |
| Zeldow et al., 1987 | Y | Y | Y | Y  (Y/Y) | N | Y | CD | Y | Y | N | Y | NA | Y | N | 9/13 | - | + | + | | + | | | - | | - | | **Fair**  **-** | |  |
|  |  |  |  |  |  |  |  |  |  |  |  |  |  |  |  |  |  |  |  | |  |  | |  | |  |  |  | |

*Abbreviations:* **CD**, cannot determine; **N**, no; **NA**, not applicable; **NR**, not reported; **Y**, yes
