# Supplementary material for: The effects of sex and gender attributes on clinical outcomes: a systematic review
Source: Biol Sex Differ. 2025 Dec 29;16:108. doi: 10.1186/s13293-025-00772-x (PMC12751436; doi:10.1186/s13293-025-00772-x)
Supplement: Supplementary file 6 — Supplementary Material 6. [file 13293_2025_772_MOESM6_ESM.docx]

**Supplement 6. Reviewer disagreement & resolution (Quality Appraisal – March 12 2025)**

| Criteria | Article | Reason for disagreement | Resolution |
| --- | --- | --- | --- |
| Criteria 2 | Moller-Leimkuhler, 2010  And  Zeldow et al., 1987 | Error checking (N vs Y) | Reviewers confirmed that rating should by Y based on article description of study population |
| Criteria 3 | N/A - multiple studies | Different interpretations of the criteria and definition of ‘eligible persons’ | Resolved via discussion and agreed on mutual definition of ‘eligible population’ as the entirety of the initially sampled population |
| Criteria 8 | Iwamoto et al., 2018 | Error checking (Y vs. N) | Reviewers agreed that rating should be Y (article reported outcomes in relation to multiple categories of exposure) |
| Criteria 9 | Yang et al., 2018 | Different interpretation of criteria (CD vs Y) | Resolved when reviewers looked at the article together and discussed and agreed on requirements for measurement tool validity going forward. |
| Criteria 14 | Snyder et al., 2016 | Error checking (N vs Y) | Reviewers agreed that rating should be Y based on authors description of age as a confounding variable |
