# Supplementary material for: The effects of sex and gender attributes on clinical outcomes: a systematic review
Source: Biol Sex Differ. 2025 Dec 29;16:108. doi: 10.1186/s13293-025-00772-x (PMC12751436; doi:10.1186/s13293-025-00772-x)
Supplement: Supplementary file 7 — Supplementary Material 7. [file 13293_2025_772_MOESM7_ESM.docx]

Supplement 7. Summary of all included studies investigating effect of sex or gender attributes on clinical outcomes

| Author (year);  Journal;  Country; Region;  City; Location of research; Study Quality (Fair, Good, Excellent) | 1. Sex and/or gender scores 2. Outcome(s) scores 3. Results of statistical analysis |
| --- | --- |
| 1. Arcand M, et al. (2023); *Front Psychol*; Canada; Quebec; Montreal; Community; Fair | 1. BSRI-SF fem/masc mean(SEM): 5.73(.06) /4.5(.07); **F**, 5.78(.07) /4.41(.09); **M**, 5.64(.1) /4.68(.1) 2. DASS-21 t_1_ /t_2_ /t_3_ /t_4_ mean(SEM): Anx 4.75(.45) /4.09(.44) /4.26(.43) /4.91(.46); Stress 10.79(.61) /10.78(.66) /11.08(.7) /11.94(.75); Depr 6.37(.57) /5.46(.51) /5.85(.53) /7.14(.62) **F**, Anx 5.29(.55) /5.16(.59) /5.48(.58) /5.35(.57); Stress 11.73(.77) /12.22(.86) /12.81(.87) /13.03(.93); Depr 6.69(.76) /6.14(.7) /6.67(.7) /7.43(.79). **M**, Anx 3.65(.74) /1.96(.46) /1.76(.35) /3.95(.74); Stress 8.86(.94) /7.92(.83) /7.57(.98) /9.53(1.15); Depr 5.73(.8). /4.12(.57) /4.16(.69) /6.51(.97) 3. Linear model:  **Anx**: main effect of sex [F_(1, 126)_=12.73, CI_95%_=1.21-4.32, *p*=.001], NS effects of time (*p*=.745), fem (p=.719), masc (*p*=.475*).* Time*sex*fem [F_(1, 376)_=3.79, CI_95%_=−1.81 to 0.02, *p*=.052].  **Stress**: main effect of sex [F_(1, 140)_=12.09, CI_95%_=1.67 to 6.23, *p*=.001], trend for time [F_(1, 386)_=2.99, CI_95%_=−.05 to .80, *p=*.085]. NS effects of fem (*p*=.95), masc (*p*=.48). Sig time*masc [F_(1, 387)_=3.86, CI_95%_=−1.93 to .12, *p*=.050], time*sex*fem [F_(1, 389)_=3.58, CI_95%_=−2.54 to .06, *p*=.059]. **Depr**: trend of time [F_(1, 384)_=3.11, CI_95%_=−.04 to .69, *p*=.079], NS effects of sex (*p*=.213), fem (*p*=.655), masc (*p*=.113).  Post-hoc: F w/ high fem had sig **↑** stress at T1, compared to M w/ high fem scores (*p*≤0.05). F w/ low fem scores had sig **↑** anx scores than M w/ low fem scores (*p*≤0.05). |
| 1. Boeri L, et al. (2017); *J Sex Med;* Italy; Milan; Academic hospital; Good | 1. TT_norm_ > 3 ng/mL; TT_low_ < 3 ng/mL; cFT_norm_ > 65 pg/mL; cFT_low_ < 65 pg/mL 2. Mean±SD, TT_norm_+cFT_norm_ / TT_norm_+cFT_low_ / TT_low_+cFT_norm_ / TT_low_+cFT_low_: IIEF-EF, 17.5±8.4 /12.6±7.7 /15.7±11.1 /12.6±8.7; IIEF-SD, 7.1±2.0 /6.1±1.9 /6.79±2.7 /6.1±2.3; IIEF-OF, 7.6±3.1 /5.7±3.6 /7.0±3.7 /5.6±3.4; IIEF-IS, 7.1±4.6 /6.2±3.9 /6.7±5.0 /6.8±4.5; IIEF-OS, 5.4±2.6 /4.9±2.6 /5.8±3.1 /5.1±2.7; BDI, 6.8±6.9 /10.6±5.5 /8.0±3.7 /10.7±7.2 3. Univariable regression (IIEF-EF/IIEF-SD/IIEF-OF/IIEF-IS/IIEF-OS/BDI): **TT_norm_+cFT_norm_** (ref); **TT_norm_+cFT_low_** (β=-3.18 [*p=*.001] CI_95%_=-7.87 to -.94 / β=-1.15 [*p=*.01] CI_95%_= -1.73 to -.25 / β=-1.18 [*p=*.001] CI_95%_= -2.9 to -.73 / NS [*p*=.32] / NS [*p*=0.43] / β=2.21 [*p=*.003] CI_95%_= 1.26 to 6.21); **TT_low_+cFT_norm_** (NS [*p*=.27] / NS [*p*=.466] / NS [*p*=.38] / NS [*p*=.74] / NS [*p*=.41] / NS [*p*=.44]); **TT_low_+cFT_low_** (β=-3.21 [*p<*.001] CI_95%_= -7.1 to -2.23 / β=-1.18 [*p=*.002] CI_95%_= -1.56 to -.34 / β=-1.23 [*p<*.001] CI_95%_= -2.84 to -.98 / NS [*p*=.07] / NS [*p*=.06] / NS [*p*=.2]; **age** (NS [*p*=.2] / NS [*p*=.2] / β=-.03 [*p=*.029] CI_95%_= -.06 to -.0 / NS [*p*=.07] / NS [*p*=.07] / NS [*p*=.06] / NS [*p*=.2]); **BMI** (NS [*p*=.2] / NS [*p*=.8] / NS [*p*=.7] / NS [*p*=.2] / NS [*p*=.6] / NS [*p*=.4]); **CCI** (NS [*p*=.08] / NS [*p*=.7] / NS [*p*=.12] / NS [*p*=.12] / NS [*p*=.2] / NS [*p*=.066]). Multivariable regression (IIEF-EF/IIEF-SD/IIEF-OF/IIEF-IS/IIEF-OS/BDI): **TT_norm_+cFT_norm_** (ref); **TT_norm_+cFT_low_** (β=-3.97 [*p=*.02] CI_95%_= -7.31 to -0.64 / β=-1.03 [*p=*.013] CI_95%_= -1.85 to -.22 / β=-1.14 [*p=*.046] CI_95%_= -2.36 to -.7 / β=-1.87 [*p=*.032] CI_95%_= -3.57 to -.17 / β=.93 [*p=*.074] CI_95%_= -1.95 to .09 / β=2.89 [*p=*.047] CI_95%_= 2.15 to 7.51); **TT_low_+cFT_norm_** (NS [*p*=.8] / NS [*p*=.4] / NS [*p*=.7] / NS [*p*=1] / NS [*p*=.3] / NS [*p*=.5]); **TT_low_+cFT_low_** (β=-3.39 [*p=*.015] CI_95%_= -6.12 to -.66 / β=-1.79 [*p=*.021] CI_95%_= -1.45 to -.12 / β=-1.46 [*p=*.004] CI_95%_= -2.46 to -.46 / NS [*p*=.2] / NS [*p*=.3] / β=3.22 [*p=*.029] CI_95%_= .3 to 6.1); **age** (NS [*p*=.8] / NS [*p*=.3] / NS [*p*=.2] / NS [*p*=.1] / β=.03 [*p=*.043] CI_95%_= 0 to .05 / β=-.11 [*p=*.01] CI_95%_= -.1 to -.02); **BMI** (NS [*p*=.7] / NS [*p*=.3] / NS [*p*=.12] / NS [*p*=.3] / NS [*p*=.5] / NS [*p*=.6]); **CCI** (NS [*p*=.4] / NS [*p*=.2] / NS [*p*=.7] / NS [*p*=.2] / NS [*p*=.3] / NS [*p*=.2]). |
| 1. Cunningham ML, et al. (2020); *Body Image*; Australia; New South Wales; Sydney; Community; Good | 1. MGRDSS mean±SD 15.59±6.58 (range 5-35) 2. MDDI mean±SD 29.85±8.95 (range 13-65); DERS mean±SD, range 3-15 (awareness 7.55±2.6, clarity 6.8±2.7, goals 9.04±3.19, impulse 5.95±2.89, strategies 6.56±2.98, nonacceptance 6.94±2.97) 3. Zero-order correlations w/ masc discrepancy stress (awareness NS [*p*=.156], clarity β=.22 [*p*<.001], goals β=.23 [*p*<.001], impulse β=.25 [*p*<.001], strategies β=.34 [*p*<.001], nonacceptance β=.31 [*p*<.001], MDDI β=.45 [*p*<.001] |
| 1. Dumesic DA, et al. (2019); *J Clin Endocrinol Metab*; USA; California; Los Angeles; Academic hospital; Good | 1. TT (ng/dL) control 29.5±2, PCOS 63.4±5.2; free T (pg/dL) control 2.3±.3, PCOS 6.0±.5 2. Adipose-IR (pmol/L x mmol/L) control 18.3±1.6, PCOS 31.9±3.8 3. Correlation w/ adipose-IR (control+PCOS): TT R=.52, R^2^=.27, *P*=.004; free T R=.61, R^2^=.38, *P*<.001 |
| 1. Gibson PA, et al. (2016); *J Affect Disord*; USA; Community; Good | 1. BSRI fem/masc mean±SD: 5.68±1.04 /4.87±.95; **F**, 5.89±.96 /4.86±.95; **M**, 5.39±1.07 /4.89±.96 2. CES-D mean±SD: 4.51±4.04; **F**, 4.95±4.23; **M**, 3.88±3.65 3. Negative binomial regression analysis, **fem** (F depr b=-.27, *p*<.001; M depr b=-.09, *p*<.001); **masc** (F depr NS, *p*>.05); M depr b=-.05, *p*<.05); F*education*fem (NS, *p*>.05); F*education*masc (b=-.08, *p*<.05); M*education*fem (b=-.11, *p*<.01); M*education*masc (NS, *p*>.05) |
| 1. Helgeson VS. (1991); *Psychosom Med;* USA; Colorado/New York; Denver/Long Island; Hospital; Fair | 1. Descriptive data NR 2. Descriptive data NR 3. Regression analysis: **perceived health** (sex NS; masc NS; hypertension β=-.022, *p<*.05; Peel index β=-.31, *p*<.01, spouse disclosure β=.21, *p*<.05); **chest pain** (sex NS; masc β=.37, *p*<.001; Peel index NS; spouse disclosure β=-.25, *p*<.01) |
| 1. Hunt K, et al. (2006); *Soc Psychiatry Psychiatr Epidemiol;* United Kingdom; Scotland; Glasgow; Community; Good | 1. Descriptive data NR 2. %SI (CI_95%_): **1930**, 5.4% (4.0-7.3), M 5.6% (3.6-8.7), F 5.3% (3.5-7.9); **1950**, 6.4% (4.8-8.4), M 3.9% (2.3-6.6), F 8.3% (6.0-11.3); **1970**, 12.3% (10.0-15.0), M 9.7% (6.9-13.6), F 14.5% (11.2-18.7) 3. Logistic regression analysis: masc/fem, 1930 SI (NS, *p*=.124 /NS, *p*=.936); 1950 SI (OR .64, CI_95%_[.46-.91], *p*=.013 /NS, *p*=.614); 1970 SI (NS, *p*=.56 /NS *p*=.731) |
| 1. Hunt K, et al. (2007); *Int J Epidemiol*; United Kingdom; Scotland; Glasgow; Community; Good | 1. BSRI fem/masc mean±SD: **F**, 5.63±.76 /4.08±.97; **M**, 5.3±.77 /4.5±.92 2. CHD mortality: **M**, 12.5%; **F**, 4.8% 3. Cox regression model, CHD mortality: **M,** masc (NS, *p*=.95), fem (HR=.68, CI_95%_[.5-.91], *p*=.011); **F**, masc (NS, *p*=.608), fem (NS, *p*=.534) |
| 1. Iwamoto D, et al. (2018); *Am J Mens Health;* USA; Maryland; College Park; Community; Good | 1. CMNI-29 mean±SD: playboy 3.1±2.21; self-reliance 4.11±1.68; violence 6.26±2.4; heterosexual 8.88±2.42; winning 6.77±2.24; risk taking 4.5±1.39; emotional control 4.52±1.87; power over women 2.81±1.61 2. BDI mean±SD: 5.88±7.83 3. Negative binomial regression: **depr,** playboy (β=.06, SE=.28, IRR=1.06, CI_95%_[1.0-1.12], *p*<.05); self-reliance (β=.22, SE=.03, IRR=1.25 CI_95%_[1.17-1.34], *p*<.01); violence (β=.06, SE=.03, IRR=1.07, CI_95%_[1.01-1.12], *p*<.05); heterosexual (NS, *p*>.05); winning (β=-.07, SE=.03, IRR=.94, CI_95%_[.89-.99], *p*<.05); risk-taking (NS, *p*>.05); emotional control (NS, *p*>.05); power over women (β=-.11, SE=.04, IRR=.90, CI_95%_[.83-.97], *p*<.01) |
| 1. Kerr P, et al. (2021); *J Psychosom Res;* Canada; Quebec; Montreal; Community; Good | 1. BSRI fem/masc mean (SE): 5.98 (0.04)/4.56 (0.05); **F**, 6.02 (0.05)/4.52 (0.06); **M,** 5.85 (0.07)/4.67 (0.09) 2. BDI-II mean (SE): 7.25 (0.57); **F,** 7.77 (0.69); **M,** 5.90 (0.97); PTSD-CC mean (SE): 33.72 (0.96); **F,** 34.64 (0.96); **M**, 31.25 (1.68) 3. Structural equation model: **depr,** masc (β=-.14, z=-2.16, *p*=.03), fem (β=-.15, z=-2.27, *p*=.02); **trauma**, masc (β=-.14, z=-2.01, *p*=.052), fem (NS, *p*=.66) |
| 1. Leinonen JT, et al. (2023); *Commun med;* Finland; Community; Good | 1. Descriptive data NR 2. Descriptive data NR 3. PGS analysis: Free T, **F** (PCOS, HR=1.02, *p*=2.8e-06; breast cancer, HR=1.04, *p*=.0001; hirsutism, HR=1.45, *p*=2.7e-08; PMB, HR=1.04, *p*=.00032); **M** (prostate cancer, HR=1.03, *p=*.0083; osteoporosis, HR=.89, *p*=.0023). MR Egger analysis: Total T, **F** (PCOS, GCP=.54, *p*=.0017; PMB, β=.61, *p*=4.5e-05; hirsutism, β=2.11, *p*=.002) |
| 1. Möller-Leimkühler A, et al. (2009**)**;  *J Affect Disorders;*  Germany; Munich; School; Good | 1. GEPAQ + masc scores: Total= 33.49 (6.03) M= 34.67 (5.80), F= 32.28 (6.03), GEPAQ + fem scores: Total= 36.64 (5.34), M=35.48 (5.16), F= 37.82 (5.20), GEPAQ - masc scores: Total= 23.28 (10.04), M= 24.67 (10.96), F= 22.25 (8.82) & GEPAQ – fem scores: Total= 20.31 (5.139), M= 19.19 (4.73), F= 21.44 (5.27) 2. GSMD total score for M= 8.27 (6.32), F= 9.64 (6.72). GSMD total score >=13: M= 18.01 (4.4), F= 18.11 (4.90); WHO-5 for M= 54.90 (20.36) F= 55.44 (20.67) 3. Multivariate ANOVA indicates binary sex & GRO had indep effects on risk of male depr w/o any interacting effects (sex: F=7.28; p=0.007; gender-role orientation: F=26.96; p=0.000)   Pearson’s R correlation btn: GEPAQ + masc & GSMD total score: -0.37, p=0.000; GEPAQ + fem & GSMD total score: -0.12, p=0.026; GEPAQ - masc & GSMD total score: 0.09, p=0.000; GEPAQ – fem & GSMD total score: 0.45, p= 0.000 |
| 1. Nguefack H, et al. (2022); *Frontier Pain Res*; Canada; Quebec; Montreal; Community; Excellent | 1. Distrib (%), F= masc/fem/adg/UD, 18.61/24.36/31.09/25.94; M= 25.28/12.36/28.65/33.71 2. # of severe adv. effects: M= 1.42 (1.96), W=1.57(2.12), fem= 2.07(2.42), masc= 1.09(1.73), adg= 1.46(2.04), UD= 1.45(2.05) 3. Post-hoc analyses: # of severe adv. effects reported: Fem-Masc, Fem-Adg, Fem-UD, sig. (p<0.0001)   Most freq. Reported adv, effects- fatigue: Fem-Adg, sig, (0.0170); dry mouth: Fem-Adg, Fem-UD, sig, (p= 0.0064); drowsiness, NS; ↓ sex drive: Fem-Masc, Fem-Adg, sig, (p=0.0029)  Most freq. severe adv. effects reported- fatigue: Fem-Masc, Fem-UD, sig, (p= 0.0004); dry mouth: Fem-Masc, Fem-Adg, sig, (p=0.0004); insomnia: Fem-Masc, Adg-Masc, sig, (p=0.0096), ↓ sex drive: Fem-Adg, Adg-UD, sig, (p=0.0022)  Model w/o interaction: GI (M vs F): -0.32, CI_95%_ = -0.52 to –0.11, sig (p= 0.0024); Gender vs UD: fem= 0.06, CI_95%_ =-0.11 to 0.23, NS (p=0.4821), masc=0.03, CI_95%_ = -0.19 to 0.25, NS (p=0. 7938), adg= 0.26, CI_95%_ = 0.09 to 0.44, sig (p=0.0030) Model w interaction: GI (M vs F): -0.58, CI_95%_ = -1.03 to –0.13, sig(p=0.0121), Gender vs UD: fem= -0.03, CI_95%_ = -0.21 to 0.16, NS(0.7788), masc= 0.03, CI_95%_ = -0.20 to 0.26, NS (p=0.8000), adg, CI_95%_ =0.08 to 0.45, sig(p=0.0042) Interaction terms: GI * Fem= 0.70, CI_95%=_ 0.16 to 1.23, sig(p= 0.0106); GI* masc= 0.09, CI_95% = −0.61 to 0.78, NS(p=0.8052), GI* adg= 0.03,_ CI_95% = -0.52 to 0.59, NS(p=0.9035)_ |
| 1. Po Yee Lo I, et al. (2019);  *Arch Sex Behav;*  United Kingdom; Oxford; USA; Louisiana; Texas; Arlington; Victoria; Abbotsford; Community; Excellent | 1. Descriptive data NR 2. Descriptive data NR 3. ANOVA, mean scores: masc, depr=4.76 (±2.77); fem, depr= 4.97 (± 3.19); Adg, depr=4.23(± 2.95); UD, depr= 6.27(3.73); Path model, direct effect: (V_1_*→*V_2_)=(β, SE, t, p-value), Masc → depr=(.06 .06 1.09 .28, NS); Fem→ depr=(-.10, .06, − 1.75, .08); Adg→ Depr(− .00, .05, − .05, .96, NS) |
| 1. Short S, et al. (2023);  *Midwifery;*  United Kingdom; Community; Good | 1. CMNI total mean±SD for emotional control, self-reliance, primacy of work, power over women: 40.29(±7.59), 15.46(±3.98), 12.06(±3.04), 7.10 (±2.05), 5.66 (±1.55); 1^st^ - time fathers mean±SD: 41.21 (±6.76),15.10 (±3.53), 12.43 (±2.77), 7.17 (±2.19), 5.63 (±1.45); 2^nd^ - time fathers mean±SD: 38.94 (±8.56),14.69 (±4.49),11.54 (±3.36), 7.00 (±1.85), 5.71 (±1.71) 2. EPDS total mean±SD: 8.86 (±5.04); 1st -time fathers mean±SD: 8.37 (±5.02); 2^nd^-time fathers mean±SD: 9.57(±5.03); MSPSS scores (mean±SD) for total= 64.58(11.79), 1^st^ time fathers= 65.31(11.11), 2^nd^-time fathers=63.50(12.77) 3. Partial correlations b/w CMNI & EPDS (r=.243, p=.09), Partial correlations b/w CMNI subscales & EPDS: Self-reliance (r=.289, p=.002), primacy of work (r=.215, p=.02), emotional control (r=.079, NS), power over women (r=.132, NS) |
| 1. Snyder PJ, et al. (2016); *New Engl J Med;* USA; Pennsylvania; Philadelphia; Hospital; Good | 1. TT, ng/dL (baseline): P group=236±67, Tx group=232±63; free T, pg/mL (baseline): P group=65±23.4, Tx group=62±21.4 2. PDQ-Q4 (baseline, change from baseline at 3mos, 6mos, 9mos, 12mos), P group (1.5±1.4, 0±1.2, -0.1±1.3, -0.1±1.3, -0.1±1.4), Tx group (1.5±1.3, 0.7±1.3, 0.6±1.6, 0.6±1.6, 0.3±1.7); FACIT-fatigue (baseline, change from baseline at 3mos, 6mos, 9mos, 12mos), P group (36.8±8.8, 4.1±9, 2.8±9, 3.7±9.2, 3.6±9.5), Tx group (37±8.6, 4.7±8.5, 4.8±8.7, 5.2±9.1, 4.7±8.8) 3. effect size, CI_95%_, p-value): Tx group (0.45, 0.33-0.58, p<.001); FACIT-Fatigue (effect size, CI_95%_, p-value): Tx group (.15, .04-.25, p=.006) 4. Random effect models for all T trials, Tx group only: Tx effect(CI_95%_), effect size(CI_95%_), p-value: PDQ-Q4= 0.62(0.45-0.79), 0.45(0.33-0.58), <0.001; FACIT-fatigue scale: 1.27(0.37-2.16), 0.15(0.04-0.25), 0.006 |
| 1. Vafaei A, et al. (2016);  *PLOS One;* Canada; Ontario, Kingston; Quebec, Montreal Brazil; Rio de Grande Norto; Community; Excellent | 1. BSRI (n, %) for masc/fem/adg/UD: M= 245(26), 136 (14.4), 294 (31.2), 267 (28.4); F= 153(14.9), 277(27), 335(32.7), 260(25.4) 2. CES-D ≥16 (n, %): M=116 (12.3), F=268 (26.1), Total=384 (19.5)   CES-D <16 (n, %): M= 826 (87.7), F= 757 (73.9), Total= 1,583 (80.5)   1. Multivariate analyses: Depr. Prevalence ratio (CI_95%_), GR (UD/fem/adg) unadjusted: 1.47 (1.15–1.88)/1.00 (0.75–1.30)/0.67 (0.51–0.90); GR (UD/fem/adg) adjusted by binary sex: 1.35(1.06 -1.71)/0.81 (0.61–1.07)/0.60 (0.45–0.80); GR (UD/fem/adg) adjusted by covariates: 1.22(0.98-1.52)/0.83 (0.64–1.07)/0.72 (0.55–0.93) |
| 1. Yang X, et al. (2018);  *J Affective Disorders;* China; Hong Kong; Shenzhen; Community; Good | 1. MGRDSS mean±SD: 2.20 (±1.08) 2. SCS mean±SD: 1.30(±.54), CESD-10 mean±SD: 0.55(±0.23) 3. Pearson correlations: MRD*social anx (β=.16, p<.01), MRD*depr (β=.17, p<.01) |
| 1. Zeldow PB, et al. (1987); *J Pers Assess;* USA;  Illinois; Chicago; Community; Fair | 1. PAQ mean±SD: masc = 21.86 (±4.42), fem=23.65(±3.75) 2. BDI mean±SD:7.85(7.39); 3. Correlations: masc*depr (-0.19, NS); fem*depr (.05, NS) |

*Note:* In this table we have used the terms ‘sex’, ‘male’ & ‘female’ when researchers reported results based on biological attributes of their participants, regardless of the term used in the original text. The use of the term ‘primary predictor’ does not imply causality but identifies the predictor as a main factor possibly associated with an outcome.

*Abbreviations:* **Adg**, androgynous; **ANOVA**, analysis of variance; **Anx**, anxiety; **BDI**, Beck Depression Inventory; **BMI**, body mass index; **BP**, blood pressure; **BSRI-SF**, Bem Sex Role Inventory-Short Form; **BSRI**, Bem Sex Role Inventory; **CCI**, Charlson Comorbidity Index; **CES-D**, Center for Epidemiological Studies-Depression Scale; **CESD-10**, Center for Epidemiological Studies-Depression Scale, Short Form; **cFT**, calculated free testosterone; **CHD**, coronary heart disease; **CI**, confidence interval; **CMNI**, Conformity to Masculine Norms Inventory; **CP**, chronic pain; **CVD**, cardiovascular disease; **DASS-21**, Depression, Anxiety, and Stress Scale-21; **Depr**, depression; **DERS-SF**, Difficulties in Emotion Regulation Scale-Short Form; **Dx**, diagnosis; **Dysreg**, dysregulation; **ED**, erectile dysfunction; **EPDS**, Edinburgh Postnatal Depression Scale; **F**, females; **FACIT-Fatigue**, Functional Assessment of Chronic Illness Therapy-Fatigue Scale; **Fem**, femininity; **GE-PAQ**, German Extended Personal Attributes Questionnaire; **GI**, gender identity; **GR**, gender role; **GRO**, gender role orientation; **GSMD**, Gotland Scale for Male Depression; **HADS**, Hospital Anxiety and Depression Scale; **HR**, hazard ratio; **Hx**, history; **IIEF-EF**, International Index of Erectile Function-Erectile Function; **IIEF-OF**, International Index of Erectile Function-Orgasmic Function; **IIEF-SD**, International Index of Erectile Function-Sexual Desire; **IIEF**, International Index of Erectile Function; **Inc**, including; **Indep**, independent; **IR**, insulin resistance; **M**, males; **Masc**, masculinity; **MBI**, Maslach Burnout Inventory; **MD**, muscle dysmorphia; **MDDI**, Muscle Dysmorphic Disorder Inventory; **MetS**, Metabolic Syndrome; **MGRDSS**, Masculine Gender Role Discrepancy Stress Scale; **MH**, mental health; **MI**, myocardial infarction; **Mod**, moderate; **Mos**, months; **MR**, Mendelian randomization; **MRD**, masculine role discrepancy; **MSPSS**, Multidimensional Scale of Perceived Social Support; **NA**, not applicable; **NB**, non-binary; **Neg**, Negative; **NR**, not reported; **NS**, non-significant; **PAQ**, Personal Attributes Questionnaire; **PCOS**, polycystic ovary syndrome; **PDQ**, Psychosexual Daily Questionnaire; **PGS**, polygenic score; **Pos**, Positive; **PP**, post-partum; **PTSD-CC**, Post-Traumatic Stress Disorder-Civilian Checklist; **Ref**, reference; **Reg**, regulation; **SCS**, Self-Consciousness Scale; **SES**, socioeconomic status; **SHBG**, sex hormone-binding globulin; **SI**, suicidal ideation; **Sig**, significant; **SRH**, self-reported health; **Sx**, symptoms; **T**, testosterone; **TD**, testosterone deficiency; **TT**, total testosterone; **UD**, undifferentiated; **VT**, vitality; **W/o**, without; **WHO-5**, World Health Organization-5 Well-Being Index; **Yrs**, years; **↑**, increased; **↓**, decreased
